# The person-based approach to intervention development: A scoping review of methods and applications

**DOI:** 10.1177/20552076241305934

**Published:** 2025-01-09

**Authors:** Lydia Holt, Sarah Denford, Hannah Bowers, Paula Kuberka, Ingrid Muller, Richard Amlôt, Lucy Yardley

**Affiliations:** 1 NIHR HPRU in Behavioural Science and Evaluation, 1980 University of Bristol, Bristol, UK; 2 School of Psychological Science, 1980 University of Bristol, Bristol, UK; 3 Primary Care Research Centre, 7423 University of Southampton, Southampton, UK; 4 Behavioural Science and Insights Unit, 371011 UK Health Security Agency, London, UK; 5 School of Psychology, 7423 University of Southampton, Southampton, UK

**Keywords:** Person-based approach, intervention development, behaviour change, methodology, digital health, scoping review, PPI

## Abstract

**Background:**

The person-based approach (PBA) has emerged as a prominent methodology guiding the development of digital and hybrid health behaviour change interventions over the last decade, and there is a salient need to understand its utilization.

**Objective:**

This study aims to describe which elements of the PBA have been utilised in intervention development research, for which populations, and how this has been reported.

**Methods:**

A search for intervention development papers published between 2015 and 2023 using forward citation searches was undertaken in Scopus, using two seed articles. Results are presented using frequency counts, and qualitative data were summarised using content analysis.

**Results:**

The review encompasses 239 papers. The PBA has frequently been applied in early stage development of digital interventions for adult populations, prioritising the use of qualitative methods. It has been used globally to develop, adapt, optimise and evaluate digital, hybrid and offline interventions for a wide range of contexts including primary and secondary healthcare, educational, community, and public health settings. Researchers value it as a proven method to identify user needs and preferences in order to create persuasive content.

**Conclusion:**

The PBA is most frequently linked to research undertaken to understand target populations and iteratively design content in early development phases. The PBA provides guidance on combining *evidence-, theory- and person-based* research, but these three elements are not always evident in the literature. Training focused on these elements, plus exemplar studies and use of reporting guidelines, could make this integrative work more visible in future papers.

## Background

Behaviour change interventions designed for digital delivery through eHealth (internet and communications technology) and mHealth (mobile and wireless devices)^1^ have become increasingly important over the last two decades, and the World Health Organization has endorsed these technologies as essential elements in working towards the aim of universal, sustainable and equitable health care.^2^

Various frameworks and approaches exist to guide the development of digital health interventions (DHIs) from intended, theorised aims into practicable outcomes, originating from both the fields of technology and design,^3–5^ and evidence-based health and medical literatures.^6–8^

**Figure 1.**
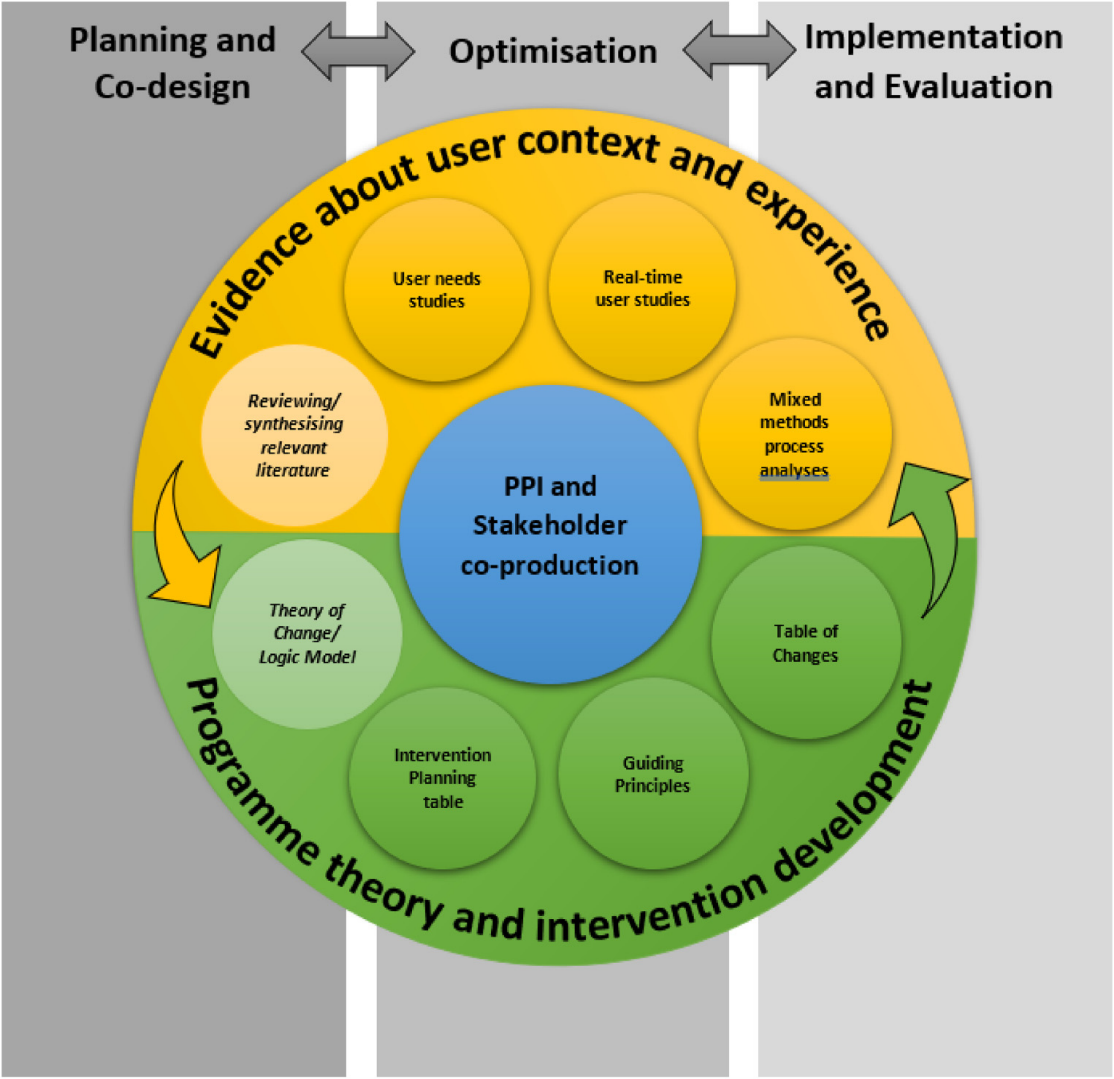
The person-based approach.

Source: https://personbasedapproach.org/index.html.

The person-based approach (PBA^9^) is a combined approach to intervention development,^10^ having been originally proposed as a framework specifically for developing DHIs which unifies existing methods from theory- and evidence-based intervention development^6,7,11^ with methods from usability testing and user-centred design.^12,13^ The rationale was to integrate approaches that should support health-related behaviour change with methods that should facilitate user engagement with a digital intervention. In order to achieve this aim, the PBA emphasizes the importance of understanding and accommodating the context and perspectives of the target population in order to deliver appropriate intervention content in an appropriate way. It encourages intervention developers to achieve this by engaging with a wide range of users at every stage of intervention development to iteratively assess and enhance the accessibility, acceptability, and feasibility of the intervention. Evidence about the beliefs, attitudes, needs, preferences, and contexts of the target users drawn from mixed-methods research and literature reviews is combined with behavioural theory and co-production with public contributors and other stakeholders to create and refine a programme theory that can guide intervention development, optimisation, evaluation, and implementation (see Figure 1).

The PBA allows the flexibility for researchers to choose from a range of knowledge generation practices and can be adapted to suit the resources and time-frame a research team has available (see Table 1). The PBA has continually evolved through reflexive adaptation as it has been applied to new contexts. For example, important methodological developments since its inception have included creating tools for collating different sources of evidence and agreeing how they should influence intervention design (see 2021 paper introducing the ‘intervention planning table’^14^), content optimisation (see 2018 paper for the ‘table of changes’^15^) and increasing co-production with public contributors and other stakeholders (discussed in a 2019 paper^16^).

**Table 1.** Elements of the PBA.

PBA element/activity	Methods can include, but are not limited to:
Literature review	Used flexibly to suit the needs of the project e.g., can use systematic, scoping, or qualitative/mixed methods synthesis to search for evidence on the effectiveness of interventions, potential content, barriers and facilitators to engagement, or relevant behaviour change theory.
User needs studies	Qualitative research such as interviews, focus-groups, or ethnography. Population level data analysis to identify specific characteristics.
Intervention planning table	Captures and summarises information from research activities, PPI and stakeholder engagement, linking this to proposed intervention content and relevant theoretical frameworks of behaviour change.
Guiding principles	These guide intervention development by specifying: (1) the key user characteristics which affect engagement, (2) a few key design objectives to improve engagement, and (3) features of the intervention which could meet the design objectives.
Real-time user studies/Think-Aloud interviews^17^	A form of qualitative interviewing whereby users engage with an intervention and are prompted to describe their experiences to the researcher in real-time. Provides very detailed feedback on accessibility and acceptability of intervention materials.
Table of changes	Tabulates feedback from activities designed to evaluate and optimise intervention content (e.g., Think-Aloud, process analysis, PPI/stakeholder discussion). Provides a coding framework to guide decision making when agreeing changes to an intervention.^5^
Patient and public/stakeholder engagement	The involvement of those who the intervention could affect, such as potential users and potential providers. This is very variable depending on the project, for example, including oversight of all research processes, participatory workshops to create content, or giving rapid feedback during optimisation. Multiple PPI approaches are recommended to maximize diversity and ensure accessibility.
Behaviour change theory	Behaviour change theory can be incorporated into the PBA via: ▪ Programme theory, explaining how the intervention is meant to achieve its goals. ▪ A logic model, summarising how the key elements of the intervention should lead to intended outcomes. ▪ The guiding principles, describing how the design supports engagement. ▪ The intervention planning table and/or the table of changes, recording why intervention elements were chosen or amended. ▪ A behavioural analysis, which links theory to the desired behaviour change, the relevant content and the intended outcomes.
Process analysis	Provides qualitative, quantitative or mixed-methods data on the how the users experience the intervention in a real-life context, and over a period of time.
Co-design/participatory research	Defined as “meaningful end-user engagement in research design”^18^ these approaches take many forms and can employ a variety of methods.
	

As yet there has been no review of the PBA literature, and currently the breadth and depth of its adoption within intervention development is unknown. Understanding the ways in which methodologies are used can help researchers better understand the approach, reflect on the strengths and weaknesses of a certain approach, guide them in choosing appropriate techniques for their own work, and create space for the discussion of best practice, with the aim of enhancing methodological rigour and reducing research waste.^19–21^ By identifying commonalities and variations in the application of the PBA in a scoping review we can move towards an understanding of how certain aspects of the PBA are used to develop, optimise, evaluate and implement behavioural health interventions. Such an understanding could then be used to inform the ongoing methodological development of the approach, create guidance and recommendations for researchers around undertaking the approach and how to report this, and influence the development of training.

In this scoping review we therefore aim to identify how research teams leverage the PBA to address the complexities of intervention development and how this has been reported, using the following review question:

How have the elements of the person-based approach been applied, and how has this been reported in papers which cite its influence on developing behaviour change interventions?

## Methods

### Protocol registration

The protocol for this review can be accessed at the Open Science Framework.^22^

### Study design

This study was informed by the foundational work of Arksey and O’Malley on scoping reviews,^23^ and the methodological guidance provided by the Johanna Briggs Institute.^24^ The PRISMA-ScR checklist^25^ has been used to ensure clarity of reporting within this paper and is included in the supplementary materials. As opposed to systematic reviews which examine the efficacy of interventions, a scoping review of methodologies does not necessarily examine the results of a study, but can be used to collate and present information on the research activities undertaken within them.^26^ Scoping reviews are concerned with broader concepts around a topic, such as identifying what kind of evidence is available, the definitions of key concepts and terminology, or how a topic has been reported on, in order to find knowledge gaps and precede further research.^27,28^

### Search strategy

Initial searches comparing keyword searches across relevant databases with forward citation searches revealed the latter strategy as the most effective in returning highly relevant references without duplication. Such a method can be employed where Boolean logic searches would fail to identify the relevant literature (e.g., due to inconsistent language and definitions, or lack of MeSH terminology).^29^ The two earliest articles outlining the PBA methodology were therefore used as seed sources for the forward citation searches^9,30^ for reports published between 1 January 2015 and 31 December 2023. These were conducted in the database Scopus, which indexed both the seed papers, and which has a high proportion of relevant journal coverage compared to Web of Science, which has lower relevant journal coverage, and did not index both seed papers.^31^

### Eligibility criteria

This paper aims to summarise author reported data of the use of the person-based approach, rather than assess if the PBA has been used ‘correctly’ against set criteria, and a broad *Population, Concept, Context* (PCC^32^) framework for inclusion was defined (see Table 2). Peer-reviewed primary research papers describing the development of an intervention, in part or in whole, in any language, were considered for inclusion in this review. Literature reviews, conference abstracts, trial registrations and posters were excluded, and papers reporting on study designs which did not specify they were undertaken as part of intervention development were ineligible.

**Table 2.** PCC framework.

*P. Population/Participants*	Any
*C. Concept*	The stated use of the person-based approach
*C. Context*	Any setting an intervention is designed for implementation within

Papers which referenced the seed papers, but which did not definitively name and state that the PBA had been influential on the methods for the development of the intervention within the narrative of the text were excluded. This approach aimed at applying inclusion/exclusion based on author-reported data. When this was ambiguous, decisions were aided by considering the placement of the citation within the paper. Papers citing seed-studies to justify their choice of methods were considered for inclusion, whilst papers citing a seed-paper as part of their discussion or future planning were excluded. Papers using synonymic variations of ‘person-based’ (e.g., ‘person-centred’ or ‘user-centred’) and citing a seed paper were considered for inclusion.

Example statement from included paper, with seed-study cited in the methods section:

This study used a rigorous person-based approach to develop a patient information leaflet for use in primary care and community pharmacy, underpinned by behavioural theory throughout interview question development, analysis, and intervention development.^33^

Example statement from excluded paper citing a seed-study in the discussion section:

…the use of digital interventions that do not fit end user values and needs could affect intervention acceptance, adoption, and diffusion, indicating that a person-centered and iterative approach is needed.^34^

### Selection procedure

Studies identified from the searches were exported into Endnote 21,^35^ and a two-stage de-duplication process was then undertaken in both SR-Accelerator,^36^ an online toolkit for systematic reviews and Rayyan,^37^ the application used for screening. The first level of screening was for inclusion on title and abstract using the PCC framework by LH, with SD and IM reviewers checking 20% of the decisions. Consensus was > 99% and resolving initial minor conflicts between two reviewers did not require the involvement of the third.

The second level of screening continued in Rayyan on full-text against the eligibility criteria. Full-text PDFs were retrieved, stored and shared in EndNote. LH undertook this with PK checking 10% of the decisions. Consensus was reached without conflict.

### Quality assessment

As a scoping review which does not report on intervention outcomes for specific populations, no critical appraisal of the relevance, value or trustworthiness of the studies was undertaken. Additionally, as a methodological study with no implications for healthcare practice or policy, it was deemed that a risk of bias assessment was not required, in line with the most recent JBI guidance.^27,38–40^

### Data extraction, analysis and presentation

A data capture form was developed, piloted and refined in the JISC online survey platform^41^ by the lead author. The research team was consulted to consider if it captured all relevant data for this study. Once all the included papers had been recorded in JISC, data was exported into Excel^42^ for cleaning and review. SD and HB checked 15% of this data against the full-text of the papers for accuracy and consistency.

Alongside metadata about the papers (e.g., title, authors, publication year), data was recorded on the target population for the intervention, the delivery of the intervention, research methods of relevance to the PBA, the use of behaviour change theories and frameworks, whether the content was novel or adapted/optimised from existing interventions, and the stages of intervention development reported by the authors, and ID frameworks and reporting guidelines used.

In order to concisely describe the extracted data initially coded categories were reviewed by the study team and collapsed into higher order groups. Data was analysed in Excel using frequency counts and pivot tables, and results are presented descriptively, with supporting tables and diagrams to visualise results. Qualitative data was analysed using content analysis via an inductive coding of the data, aiming to condense the multiple author descriptions into a fewer categories, without undue abstraction or over-interpretation^43^ (see codebook in supplementary material).

## Results

### General findings

**Figure 2.**
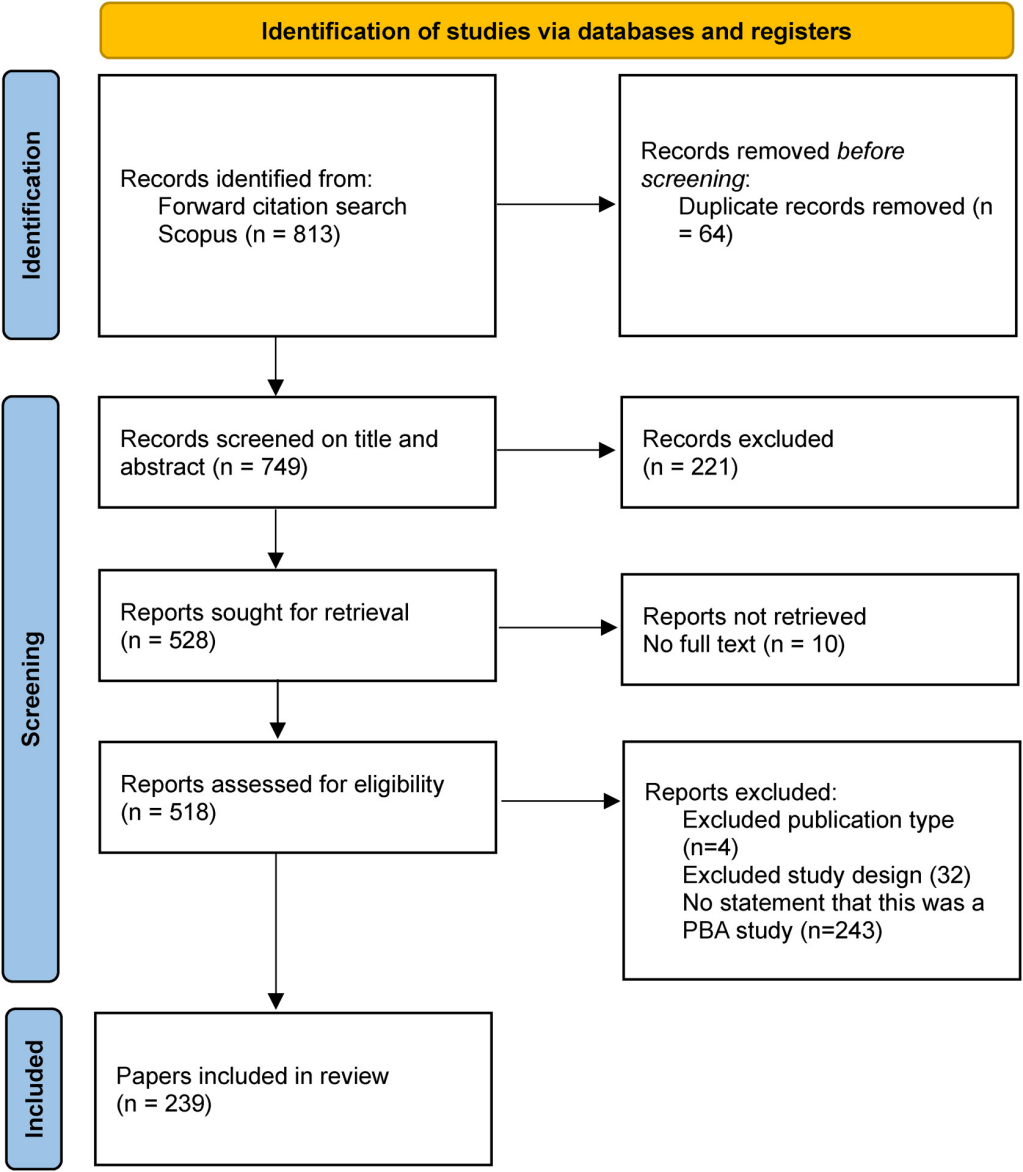
PRISMA flow diagram for selection of papers.

After searching the literature for narrative descriptions of using the PBA to develop health interventions, 239 reports have been identified, shown in the PRISMA-ScR flow diagram^44^ in Figure 2. The full list of papers can be found in the supplementary material, and all data is reported at the paper level although some papers are linked (e.g., multiple reports of one study or intervention). This is due to the ways that development papers are often split and published as work progresses, and methods may be used more than once for multiple intervention components (e.g., Think-Aloud interviews might be undertaken for both content designed for patients and content designed for health care providers). As part of the focus of this paper is on how researchers have reported on their use of the PBA, summarising the review at paper level also allows the data to be looked at for year-on-year trends.

#### Papers per year, and types of report

**Figure 3.**
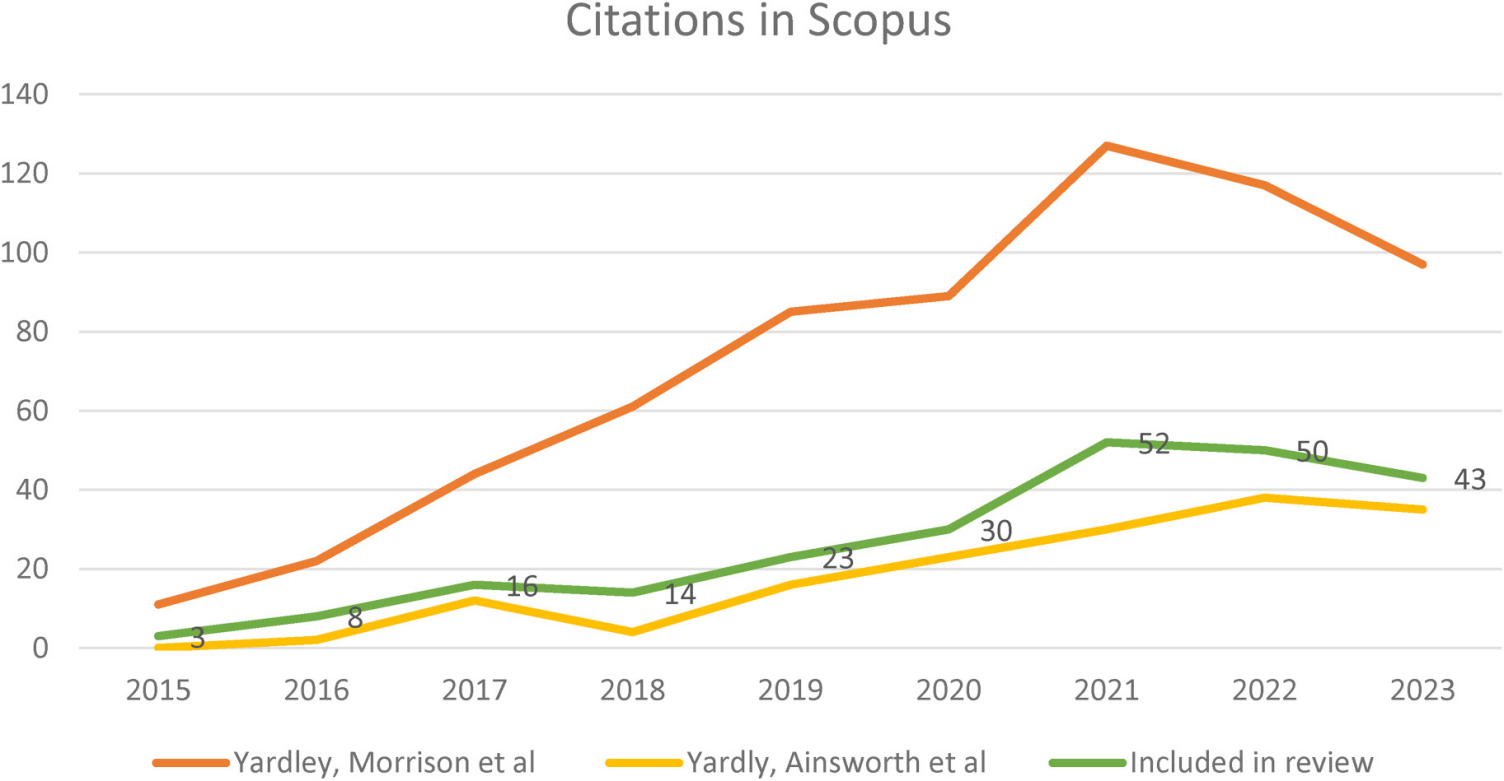
Citing and included papers per year.

There has been a steady growth in the influence of the PBA, as indicated by the rising number of citations of the 2015 methods papers^9,30^ in Scopus each year since their publication (Figure 3). In the paper included in this review, 138 of these papers were categorised as intervention development (ID) papers as they primarily focused on part of, or the overall development of a specified intervention programme. Thirty-three were protocol papers, 26 were papers focusing on pilot or feasibility findings, 20 primarily reported the study results e.g., a randomised control trial (RCT), and 19 focused on the evaluation of an intervention (e.g., process evaluations of RCTs). Three were qualitative papers which stated the research undertaken was to inform intervention development but did not specify a particular study.

##### Country of study setting

**Figure 4.**
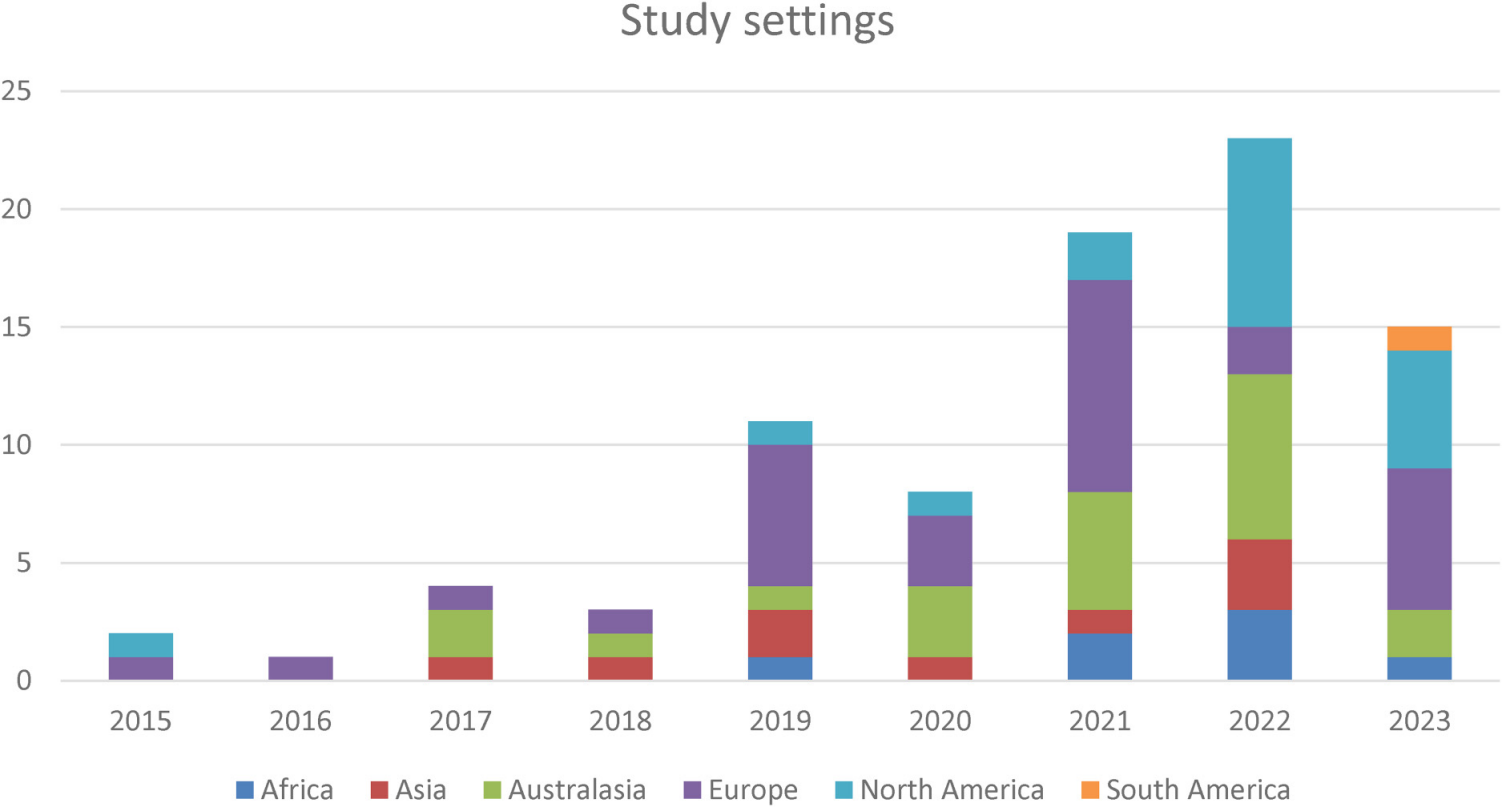
Continents of study settings by year of publication.

*Note*. The above excludes the UK from the data for clarity of reporting. Some papers reported >1 setting.

The majority of the papers reported on studies set in the UK (*n = *161), however, as publications increased, so too did the reporting of study locations outside of the UK. Figure 4 shows the number of reported settings by continent, excluding the UK from the count for clarity of visualisation. Overall, 29 different countries were reported as study settings, with Europe as the most frequently reported continent (*n = *30), followed by studies set in Australasia (*n = *21).

### Target population

The most frequent aim reported in the included papers was to develop interventions intended to benefit adult populations defined by communicable and non-communicable physical health conditions, such as diabetes, hypertension, infections, injury, or cancer (*n = *146). These interventions targeted physical outcomes (e.g., blood glucose levels) and/or psycho-social experiences related to the health condition (such as anxiety or depression). 53 papers were categorised as reporting targeting populations defined by mental health conditions, with the criteria for inclusion in this category that the primary target of the intervention is listed within the Diagnostic and Statistical Manual of Mental Disorders (DSM-5-TR^45^). 40 of the papers reported targeting populations without a defined pathology, such as general population or workplace health promotion, or did not fit into discreet categories such as the implementation of administrative procedures in healthcare settings, or improving athletic performance. Adults over the age of 18 were the most frequent target population, however older adults were the least frequently reported group (see Table 3).

**Table 3.** Ages of target populations, as reported in individual papers.

Population	Number of papers
Adults	176
Adolescents	18
Undefined	17
Older adults	9
Children	8
Children and adolescents	7
Adolescent and adults	4
	239

*Note*. Adults: ≥18; adolescents: ≥13 to ≤17, children: ≤12, older adults as defined within each paper. Undefined: assumed general population, or not reported.

20 of the papers reported intervention delivery via intermediaries, for example, the intervention was professional training for clinicians intended to ultimately benefit a patient population (*n = *16), or for parents and carers (*n = *3) that would benefit their child, or an employer, with the target population being the employees (*n = *1).

### Mode of delivery

One hundred and seventy-six of the papers reported developing interventions for digital delivery such as via a website, smartphone app, or digitally integrated technology, and 73 of these also featured human-interaction as a feature, for example, via therapeutic / clinical input from a health care professional or trained supporter (*n = *61), informal peer support (*n = *8), or parent, carer or teacher (*n = *4), or a combination. Fifty-three papers reported interventions delivered though non-digital means such face-to-face therapies, administrative procedures, or non-digital media such as printed handbooks. Ten papers did not describe a mode of delivery, (e.g., due to being early in the research and planning stages).

It was not always possible to categorise through what setting the programmes would reach the target population (*n = *86) and reporting of this was frequently ambiguous, especially for DHIs, therefore where participants were recruited from was often used as a proxy for data extraction purposes. In those papers coded as specifying targeting a population via a health care system, the most frequent reach to the beneficiary was made through primary care (*n = *68), with secondary care settings the next most frequent (*n = *37). Community, third-sector, workplace, educational and general population settings were also reported as means of reaching a target user or delivering an intervention (*n = *51), and some papers reported delivery could be through more than one setting.

### Use of intervention development guidance and reporting frameworks

One hundred and thirty-five papers did not narratively describe the use of development guidance other than the PBA, and the majority that did cited that Medical Research Council (MRC) guidance on the development of complex interventions was used (*n = *71).^7,46,47^ Forty-three referenced other frameworks, often reflecting the digital design and delivery of the intervention (e.g., Ritterband's framework for internet interventions,^48^ the Behavioural Intervention Technology model^49^).

When reporting the intervention development process, nine of the papers used GUIDED (Guidance for reporting intervention development studies in health research),^50^ a 14-item checklist to improve ID reporting and transparency. GUIDED was first published in April 2020 and not available to cite in around over half of the included papers. When describing the intervention design itself, 25 of the papers used the 12-item TIDieR template for intervention description and replication checklist, which was published March 2014,^51^ before the first Person-Based methodology paper and therefore available to all authors.

### Development stages and source of intervention content

Authors infrequently defined which phases of research the PBA was used for, therefore all stages of development described in each paper was coded.

The planning stage of intervention development was most frequently reported (*n = *166, 69% of all papers), compared to optimisation (*n = *118, 49% of all papers) or evaluation (*n = *92, 38% of all papers). In a small number of trial papers it was unclear what the development process had been (see Table 4).

**Table 4.** Stages of development as reported in individual papers.

Stages	Number of papers
Planning and optimising	76
Planning	47
Evaluating	44
Planning, optimising and evaluating	22
Planning, evaluating	21
Optimising	15
Not reported	9
Optimising and evaluating	5
	239

*Note*. Planning – activities up to the creation of a prototype or draft intervention content; Optimisation – refinement of prototype or draft intervention; Evaluation – test of acceptability of content, such as during a pilot, feasibility, or trial (not the effectiveness).

The use of existing intervention content, such as adaption for a new mode of delivery or population, was reported in 31% (*n = *75) of the included papers.

### Key elements of the person-based approach

**Figure 5.**
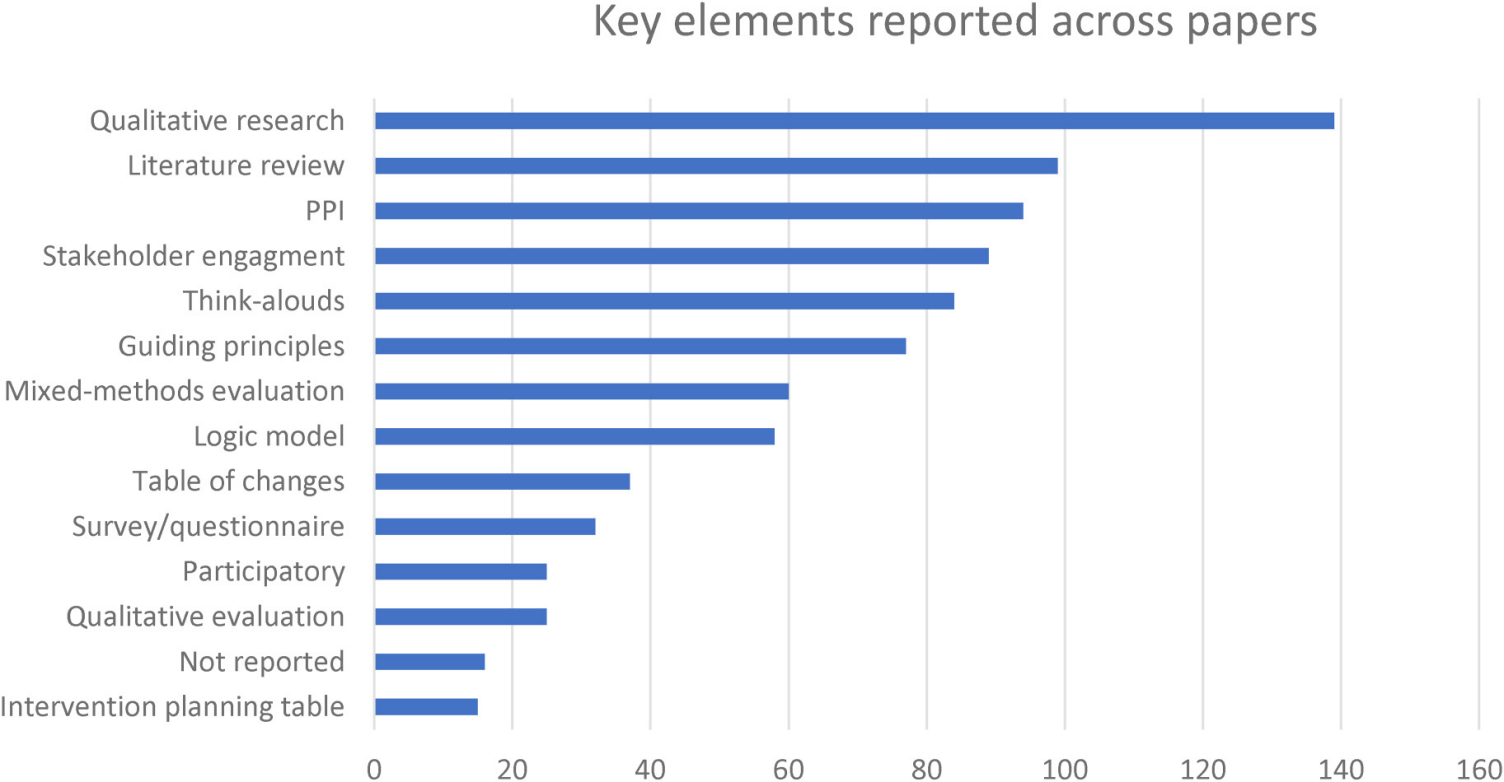
Key elements of the PBA.

*Note*. Table of changes introduced 2018, and intervention planning table introduced 2021. We used author definitions for inclusion in the category ‘participatory’.

Data were collected on all relevant methods in the included papers, however it most instances it was not possible to conclude whether these were undertaken because of fidelity to the elements recommended in the PBA (see Table 1). The most frequent key elements reported in the papers were early stage primary qualitative research (i.e., undertaken before evaluation phases, *n = *139), followed by literature reviews (*n = *99), patient and public involvement (PPI; *n = *94), and stakeholder involvement (*n = *89), see Figure 5. Think-Aloud interviews (*n = *84) and creating guiding principles (*n = *77) were the next most frequent reported activities, and these results align with this review's findings that the PBA is commonly linked to the early stage planning and optimisation phases of development.

### Use of theory

Many papers (*n = *111) did not describe using a theory-based approach to intervention development. Where theories were referenced, descriptions were often brief, lacking details on why they were chosen, how they would facilitate change, their relevance to intervention content, or their alignment with the psychosocial context of the target population. The theories cited were also often psychological models that can explain behaviours, rather than behaviour change techniques (BCTs) that can promote change. Table 5 summarizes how often influential theory-based frameworks, models, and taxonomies of behaviour change commonly used in intervention research were reported in the reviewed papers. However, these were often referenced without specifying which underlying taxonomic elements were selected to promote behaviour change.

**Table 5.** Theory-based approaches to behaviour change.

Theory-based approaches	Number of times reported
COM-B; Capability, opportunity, motivation, behaviour model^52^	33
BCW; Behaviour change wheel^52^	32
TDF; Theoretical domains framework^53^	29
BCT.v1; Behaviour change technique taxonomy (v1)^54^	28

*Note*. Papers frequently reported using more than one approach.

### Rationale for using the PBA

Rationales for using the PBA were captured verbatim from the texts, and analysed with content analysis.^43^ Three higher order codes were used to categorise author's reasons (see appendix for codebook):

***Methods focused ***– this was the most frequent reason given for using the PBA. Authors using this rationale valued the PBA as a proven, rigorous and systematic process for intervention design which guided them in the use of specific methods, such as in the example given below:

“…the person-based approach provided a systematic and robust method to combine user-centred design methods with evidence-based behaviour change methods.”^55^

***Intervention focused*** – these rationales focused on using the PBA to create content which could establish positive user interactions with intervention content, such as ‘engagement’, ‘feasibility’, ‘acceptability’ and ‘accessibility’. These were linked to a greater likelihood of achieving the intended intervention outcomes such as in the following quote, where the PBA was used

“…to ensure that the intervention is effective and acceptable to those who will ultimately use it.”^56^

***User focused***– these rationales cited the PBA as providing a way to understand user needs and preferences in both the experience of using digital content, and their wider psychosocial context. The PBA was described as a means to

“…elicit an in-depth understanding of the target user and their psychosocial context to guide the selection of key behavioural techniques in the specific context of the intervention.”^57^

## Discussion

### Main findings

This review is the first to examine the use of the person-based approach and has gathered data on its adoption and application in order to consider the current impact and future development of the methodology within health research. The results chart the increasing utilisation of the PBA to plan novel digital and hybrid health interventions for adults in the UK with defined physical or mental health needs, but also within fields other than health, and for non-digital interventions, as well as growing usage internationally. The most frequently reported key element was the evidence-generating activity of qualitative research in order to understand target population needs. The use of theory and BCTs to explain how interventions would create behaviour change was less commonly reported.

Work undertaken in the planning stage of intervention development was the most frequently reported research activity; this included undertaking literature reviews, qualitative research and PPI /stakeholder engagement. However, despite the prominence of PPI in methodological considerations of the PBA^16^ and the model itself (see ‘co-design’ and ‘PPI’ in Figure 1), under half the papers reported engaging with the target population in this way (39%), though this finding is consistent with wider searches of the health literature.^58^ Involving the people for whom research is intended to benefit is now recommended by major funders of research however, including the National Institute for Health Research (NIHR) in the UK, the Canadian Institutes of Health Research, the European Innovative Medicines Initiative, and the Australian National Health and Medical Research Council. The intended impacts of PPI include increased recruitment, retention and enrolment, and increased confidence of participants.^59^ Involvement of those for whom the research is intended to benefit should certainly be seen as a means to ensure research participation is equitable,^60^ but the aim of the PBA is to move beyond representation by making interventions engaging, feasible and useful as possible for a wide range of users, including those underserved communities who are most often at risk of poor health outcomes,^61^ and this can be achieved through careful consideration of how to integrate diverse user views into intervention development.

The paper by Rai et al. 2021 (which includes authors of the originating PBA methodology) describes optimising an intervention through deliberately sampling socio-economically and ethnically diverse participants with a variety of disabilities for qualitative research, and then integrating this with the viewpoints of their PPI panel.^62^ They report that this use of the PBA enabled them to refine content to ensure it was accessible for people with differing language abilities, family/social contexts of potential intervention users, and for varied levels of digital and health literacy. The PBA practice of using a table of changes facilitates recording this process of integration, as well as the resulting development/design decisions. There was not enough overall evidence in the included studies to assess if other researchers are using the PBA to address health and research inequities in their work, however both the underlying principles and the user-focused research activities of the approach are intended to support this aim.

When looking at how researchers most often engaged with their target populations, data from this review links the undertaking of early qualitative research with forming a fundamental understanding of the intended beneficiaries, such as via the exploration of barrier and facilitators to engagement with interventions, and to gain a deep understanding of users’ health needs and experiences. Qualitative work was most frequently undertaken upfront, though it also contributed to later evaluative activities (such as within a process analysis). This aligns with the aim of PBA activity of using evidence to create Guiding Principles early on, in order to ground the ongoing development work in the core needs of the population, and particularly in their initial engagement with intervention content.^63^

The use of the PBA was most often reported in papers which describe planning and early-stage intervention research. While the PBA can be applied usefully throughout the cycle of development, implementation and evaluation, alternative guidelines for using qualitative data to evaluate interventions are already well-established in the lierature^7,8,64^ However, testing and trialling interventions requires a large investment of time and money,^65^ and ensuring the acceptability of content by undertaking careful planning and refinement work in early development stages rather than reaching an understanding later is optimal. The PBA offers clear guidance for the testing and optimisation of content prior to feasibility or effectiveness trials, and this may explain its growth as a method within early health intervention research. This attention to the initial stages of development is particularly relevant for creating digital content, allowing for wire-framing and real-time user studies to walk users through early protypes of intervention content and gain feedback on the experience.^66^ Evaluating the impact of using the PBA for early-stage content refinement on overall research costs could be an avenue of future research.

Although all of the interventions sought to change behaviour in some way, not all of the papers described if a behaviour change technique, model, framework or theory was used to explain or promote this. Previous reviews of how theorising has been reported in the intervention design literature similarly concluded that where reported, actual detail on how theory was linked to the selection of content or mechanisms of action was insufficient, and that health interventions are frequently developed without reference to any theory.^67,68^ The process of theorising is perhaps not always easily described but when missed it limits the understanding of how behaviour change happens, and the ability to replicate this.^69^ Further to this, Davis et al.^70^ explain that the use of theory is important in the development of interventions because it helps researchers to: identify the causes of behaviours; tailor behaviour change methods to suit the target population; understand whether an intervention was ineffective due to a failure to impact the mediators of the target behaviour or because the successfully influenced mediator does not actually affect the target behaviour; and gather evidence on how to change behaviour across various populations and contexts.

It was frequently unclear, particularly for DHIs, in what setting the interventions would be implemented, or how initial reach would be made to target users. This may be because a DHI could potentially be delivered through more than one setting, however they may often be initially ‘prescribed’ within a clinical setting, and therefore development work should consider issues of delivery and integration into existing systems.^71^ Clearer reporting around how these issues were researched and addressed would aid in understanding the current context of the intervention, its intended implementation, and any future adaptation and transfer of the programme to other settings.^72^

### Current impact and future directions

This study has examined the application of one approach to intervention development, but reports results which are consistent with broader reviews of the literature, such as a lack of visibility of behaviour change theorising, or the description of PPI work. It may be that the low visibility of PPI in papers is due to a historical absence of guidance around recording and reporting these activities in comparison to more established research undertakings where intervention users are recruited as participants. Systematically logging, evaluating and reporting PPI, such as with GRIPP2,^73^ could aid in future evaluations of the breadth, depth and impact of engagement being undertaken in intervention development studies.

Clear reporting of how behaviour change theory has been leveraged to promote user engagement and adherence is particularly relevant for digital interventions.^48^ As a ‘combined’ approach^10^ to intervention development with the original full title of ‘*evidence-, theory- and person-based approach*’ the PBA is rooted in both behavioural science and user-centred design,^9^ and therefore aims to promote both upfront digital engagement and offline behaviour change. Using theory to explain user engagement at the micro (active engagement with a digital interface) and macro levels (active engagement with target behaviours)^74^ is therefore central to understanding how a digital intervention will achieve its aims.

The results of this study describe how researchers who are using the PBA to develop interventions value and prioritise the use of qualitative methods to understand user perspectives. Further methodological work may need to be done to illustrate how to use the PBA to integrate qualitative work with other elements, such as theory and PPI, and the importance of doing so. This could be addressed in future exemplar papers of the PBA, or within formal training.

As a broad scoping review of current practice this study offers a launching point for future work, such as a qualitative study with PBA researchers to determine why they have made methodological decisions; this type of study has previously been used to surface the challenges of intervention development in the face of limited resources, and in order to share best practice.^75,76^ Both effectiveness and non-health impacts of interventions should also be studied in order to prevent research waste; for the PBA such analysis could consider if rigorous and inclusive upfront development impacts overall research costs, perhaps by preventing the expensive evaluation of interventions that have not been optimised. It would also be important to consider if using the PBA helps address health inequities by revealing and responding to differing user needs. To undertake this kind of evaluative work of the approach however, clearer definitions around what a PBA intervention is would need to be made. This review suggests identifying PBA studies would be challenging however, due to variations in both the implementation and reporting of research processes, and the inherent flexibility needed to respond to different settings, contexts and populations common to all development methods.^77^ It may therefore be more appropriate to test non-health outcomes such as acceptability and accessibility via experimental methods for the future evaluation of the impacts of the PBA; this could be undertaken as part of the development process to ensure intervention content is optimised for the target population.^78^

### Limitations

This review is a sample of the literature, including only articles indexed in Scopus, reported at paper-level, and does not capture and synthesize every study which the PBA has influenced. This is because inclusion and extraction relied on unambiguous reporting in an effort to avoid over-representation, and instead use author reported data, resulting in including a much smaller number of papers than actually referenced the PBA in the intervention development literature (see Figure 1). As the criteria for both inclusion and data extraction was a clear statement of use in the narrative of the text there will be some misalignment between the actual work the authors of the included studies undertook and the coding of the papers in this review, however any underreporting by authors may in part be influenced by publication norms. Expectations of concision and linearity in scientific writing can be at odds with the iterativity of the intervention development process, and those publishing in applied fields face similar pressures as those in experimental science to “smooth over imperfections”^79^ when presenting their work.

The stages of development, and many of the research activities within the PBA are not exclusive to the approach and overlap with other methodological frameworks. Although over half of the papers only named the PBA as a development guide it is still not possible to make defined claims that certain methods were used solely due to fidelity to the approach. Where authors illustrated how other frameworks were incorporated this aided considerably in the understanding the impact this has on the selection of methods and the overall development process (see Figure 1 in Beck et. al regarding the integration of the PBA, BIT-Model and IDEAS Framework^80^ and Figure 2 in Arden et.al which details the use of distinct frameworks across different stages^81^).

Ambiguity around clearly identifying what research activities have undertaken and why points towards a need for greater detail and transparency when reporting methods for intervention development, adding evidence that the use of reporting standards such as TIDIER^51^ and GUIDED^50^ by authors could support other researchers in interpreting and learning from the evidence base.

## Conclusions

This scoping review has provided insights towards understanding the pragmatic considerations that shape research practices of intervention developers using the person-based approach. It has become well-established in the literature as a method for planning and optimising novel interventions using qualitative methods to generate evidence about user needs and preferences, particularly for digital delivery. The papers in this review cited the PBA as a valued source of guidance on how to establish target population needs within an iterative process allowing continuous appraisal in initial design phases, and the role of qualitative methods to do so. As an integrative and combined approach to development, future work on the PBA should clarify the importance of also including diverse user perspectives via PPI and participatory approaches, as well as how to make visible the underlying theories that are proposed to drive change. Further research into how the PBA influences intervention processes, equitable participation and research efficiency is therefore merited. As an emerging and dynamic development approach, the PBA will continue to evolve and be refined in response to the needs of the researchers who use it, and the populations it seeks to benefit.

## Supplemental Material

sj-docx-1-dhj-10.1177_20552076241305934 - Supplemental material for The person-based approach to intervention development: A scoping review of methods and applications Supplemental material, sj-docx-1-dhj-10.1177_20552076241305934 for The person-based approach to intervention development: A scoping review of methods and applications by Lydia Holt, Sarah Denford, Hannah Bowers, Paula Kuberka, Ingrid Muller, Richard Amlôt and Lucy Yardley in DIGITAL HEALTH

sj-docx-2-dhj-10.1177_20552076241305934 - Supplemental material for The person-based approach to intervention development: A scoping review of methods and applications Supplemental material, sj-docx-2-dhj-10.1177_20552076241305934 for The person-based approach to intervention development: A scoping review of methods and applications by Lydia Holt, Sarah Denford, Hannah Bowers, Paula Kuberka, Ingrid Muller, Richard Amlôt and Lucy Yardley in DIGITAL HEALTH

sj-docx-3-dhj-10.1177_20552076241305934 - Supplemental material for The person-based approach to intervention development: A scoping review of methods and applications Supplemental material, sj-docx-3-dhj-10.1177_20552076241305934 for The person-based approach to intervention development: A scoping review of methods and applications by Lydia Holt, Sarah Denford, Hannah Bowers, Paula Kuberka, Ingrid Muller, Richard Amlôt and Lucy Yardley in DIGITAL HEALTH
