# Supplementary material for: The person-based approach to intervention development: A scoping review of methods and applications
Source: Digit Health. 2025 Jan 9;11:20552076241305934. doi: 10.1177/20552076241305934 (PMC11719439; doi:10.1177/20552076241305934)
Supplement: sj-docx-2-dhj-10.1177_20552076241305934 - Supplemental material for The person-based approach to intervention development: A scoping review of methods and applications [file sj-docx-2-dhj-10.1177_20552076241305934.docx]

**Included papers:**

1. Adeagbo O, Kim H-Y, Tanser F, et al. Acceptability of a tablet-based application to support early HIV testing among men in rural KwaZulu-Natal, South Africa: a mixed method study. *AIDS care* 2021; 33: 494-501.

2. Ahmed A, Steed L, Burden‐Teh E, et al. Identifying key components for a psychological intervention for people with vitiligo–a quantitative and qualitative study in the United Kingdom using web‐based questionnaires of people with vitiligo and healthcare professionals. *Journal of the European Academy of Dermatology and Venereology* 2018; 32: 2275-2283.

3. Ainsworth B, Steele M, Stuart B, et al. Using an Analysis of Behavior Change to Inform Effective Digital Intervention Design: How Did the PRIMIT Website Change Hand Hygiene Behavior Across 8993 Users? *Annals of behavioral medicine : a publication of the Society of Behavioral Medicine* 2017; 51. DOI: doi:10.1007/s12160-016-9866-9 [doi].

4. Ainsworth B, Greenwell K, Stuart B, et al. Feasibility trial of a digital self-management intervention 'My Breathing Matters' to improve asthma-related quality of life for UK primary care patients with asthma. *BMJ open* 2019; 9. DOI: doi:10.1136/bmjopen-2019-032465 [doi].

5. Ainsworth B, Miller S, Denison-Day J, et al. Infection Control Behavior at Home During the COVID-19 Pandemic: Observational Study of a Web-Based Behavioral Intervention (Germ Defence). *Journal of medical Internet research* 2021; 23. DOI: doi:10.2196/22197 [doi].

6. Ainsworth B, Horwood J, Walter SR, et al. Implementing Germ Defence digital behaviour change intervention via all primary care practices in England to reduce respiratory infections during the COVID-19 pandemic: an efficient cluster randomised controlled trial using the OpenSAFELY platform. *Implementation Science* 2023; 18: 67.

7. Allan S, Mcleod H, Bradstreet S, et al. Understanding Implementation of a Digital Self-Monitoring Intervention for Relapse Prevention in Psychosis: Protocol for a Mixed Method Process Evaluation. *JMIR research protocols* 2019; 8. DOI: doi:10.2196/15634 [doi].

8. Anderson AC, Robinson AH, Potter E, et al. Development of Goal Management Training(+) for Methamphetamine Use Disorder Through Collaborative Design. *Frontiers in psychiatry* 2022; 13. DOI: doi:10.3389/fpsyt.2022.876018 [doi].

9. Anderson AM, McHugh GA, Comer C, et al. Supporting patients to prepare for total knee replacement: Evidence‐, theory‐and person‐based development of a ‘Virtual Knee School’digital intervention. *Health Expectations* 2023; 26: 2549-2570.

10. Arden MA, Hutchings M, Whelan P, et al. Development of an intervention to increase adherence to nebuliser treatment in adults with cystic fibrosis: CFHealthHub. *Pilot and feasibility studies* 2021; 7. DOI: doi:10.1186/s40814-020-00739-2 [doi].

11. Ardern CL, Hooper N, O'Halloran P, et al. A Psychological Support Intervention to Help Injured Athletes "Get Back in the Game": Design and Development Study. *JMIR formative research* 2022; 6. DOI: doi:10.2196/28851 [doi].

12. Aubut V, Goyette M, Flores-Aranda J, et al. A community-based case study of the co-construction of an online intervention with gay and bisexual men who use substances. *Journal of Community Practice* 2023; 31: 44-62.

13. Bailey PK, Ben-Shlomo Y, Caskey FJ, et al. Development of an intervention to improve access to living-donor kidney transplantation (the ASK study). *PloS one* 2021; 16. DOI: doi:10.1371/journal.pone.0253667 [doi].

14. Bailey PK, Caskey FJ, MacNeill S, et al. Investigating strategies to improve AccesS to Kidney transplantation (the ASK trial): a protocol for a feasibility randomised controlled trial with parallel process evaluation. *Pilot and Feasibility Studies* 2023; 9: 13.

15. Bamgboje-Ayodele A, Levesque JV, Gerges M, et al. The male perspective: A mixed methods study of the impact, unmet needs and challenges of caring for women with breast cancer. *Journal of psychosocial oncology* 2021; 39: 235-251.

16. Band R, Morton K, Stuart B, et al. Home and Online Management and Evaluation of Blood Pressure (HOME BP) digital intervention for self-management of uncontrolled, essential hypertension: a protocol for the randomised controlled HOME BP trial. *BMJ open* 2016; 6. DOI: doi:10.1136/bmjopen-2016-012684 [doi].

17. Band R, Bradbury K, Morton K, et al. Intervention planning for a digital intervention for self-management of hypertension: a theory-, evidence- and person-based approach. *Implementation science : IS* 2017; 12. DOI: doi:10.1186/s13012-017-0553-4 [doi].

18. Band R, Hinton L, Tucker KL, et al. Intervention planning and modification of the BUMP intervention: a digital intervention for the early detection of raised blood pressure in pregnancy. *Pilot and feasibility studies* 2019; 5. DOI: doi:10.1186/s40814-019-0537-z [doi].

19. Beatty L, Koczwara B, Butow P, et al. Development and usability testing of a web-based psychosocial intervention for women living with metastatic breast cancer: Finding My Way-Advanced. *Journal of cancer survivorship : research and practice* 2021; 15. DOI: doi:10.1007/s11764-021-01019-5 [doi].

20. Beck AK, Kelly PJ, Deane FP, et al. Developing a mHealth Routine Outcome Monitoring and Feedback App ("SMART Track") to Support Self-Management of Addictive Behaviours. *Frontiers in psychiatry* 2021; 12. DOI: doi:10.3389/fpsyt.2021.677637 [doi].

21. Bell A, Grampurohit N, Kains G, et al. Developing guiding principles for technology-based rehabilitation program by engaging people with motor incomplete tetraplegia. *Journal of neuroengineering and rehabilitation* 2022; 19. DOI: doi:10.1186/s12984-022-01096-2 [doi].

22. Bendelin N, Björkdahl P, Risell M, et al. Patients' experiences of internet-based Acceptance and commitment therapy for chronic pain: a qualitative study. *BMC musculoskeletal disorders* 2020; 21. DOI: doi:10.1186/s12891-020-03198-1 [doi].

23. Berry N, Machin M, Ainsworth J, et al. Developing a theory-informed smartphone app for early psychosis: learning points from a multidisciplinary collaboration. *Frontiers in psychiatry* 2020; 11: 602861.

24. Besenyi GM, Bramwell RC, Heinrich KM, et al. A qualitative exploration of park-based physical activity in adults with serious mental illness: Insights from peers and peer counselors. *Mental Health and Physical Activity* 2022; 23. DOI: doi:https://dx.doi.org/10.1016/j.mhpa.2022.100466.

25. Bevan Jones R, Thapar A, Rice F, et al. A Web-Based Psychoeducational Intervention for Adolescent Depression: Design and Development of MoodHwb. *JMIR mental health* 2018; 5. DOI: doi:10.2196/mental.8894 [doi].

26. Bevan Jones R, Thapar A, Rice F, et al. A Digital Intervention for Adolescent Depression (MoodHwb): Mixed Methods Feasibility Evaluation. *JMIR Ment Health* 2020; 7: e14536. 20200717. DOI: 10.2196/14536.

27. Bingham SL, Semple CJ, Flannagan C, et al. Adapting and usability testing of an eLearning resource to enhance healthcare professional provision of sexual support across cancer care. *Supportive care in cancer : official journal of the Multinational Association of Supportive Care in Cancer* 2022; 30. DOI: doi:10.1007/s00520-022-06798-w [doi].

28. Bingham SL, Semple CJ, Flannagan C, et al. Enhancing healthcare professional‐led sexual support in cancer care: Acceptability and usability of an eLearning resource and its impact on attitudes towards providing sexual support. *Psycho‐Oncology* 2022; 31: 1555-1563.

29. Birtwell K, Morris R and Armitage CJ. Brief Mindfulness-Based Interventions: Teacher and Course Attendee Perspectives on Content. *Mindfulness* 2021; 12. DOI: doi:10.1007/s12671-021-01698-2.

30. Bishop FL, Greville-Harris M, Bostock J, et al. Using psychological theory and qualitative methods to develop a new evidence-based website about acupuncture for back pain. *European journal of integrative medicine* 2016; 8. DOI: doi:10.1016/j.eujim.2016.05.006 [doi].

31. Blake H, Vaughan B, Bartle C, et al. Managing Minds at Work: Development of a Digital Line Manager Training Program. *International journal of environmental research and public health* 2022; 19. DOI: doi:10.3390/ijerph19138006 [doi].

32. Borek AJ, Campbell A, Dent E, et al. Development of an intervention to support the implementation of evidence-based strategies for optimising antibiotic prescribing in general practice. *Implementation science communications* 2021; 2. DOI: doi:10.1186/s43058-021-00209-7 [doi].

33. Bowers HM, Kendrick T, Glowacka M, et al. Supporting antidepressant discontinuation: the development and optimisation of a digital intervention for patients in UK primary care using a theory, evidence and person-based approach. *BMJ open* 2020; 10. DOI: doi:10.1136/bmjopen-2019-032312 [doi].

34. Bowers H, Kendrick T, van Ginneken N, et al. A Digital Intervention for Primary Care Practitioners to Support Antidepressant Discontinuation (Advisor for Health Professionals): Development Study. *Journal of medical Internet research* 2021; 23. DOI: doi:10.2196/25537 [doi].

35. Bradbury K, Morton K, Band R, et al. Understanding how primary care practitioners perceive an online intervention for the management of hypertension. *BMC medical informatics and decision making* 2017; 17. DOI: doi:10.1186/s12911-016-0397-x [doi].

36. Bradbury K, Morton K, Band R, et al. Using the Person-Based Approach to optimise a digital intervention for the management of hypertension. *PloS one* 2018; 13. DOI: doi:10.1371/journal.pone.0196868 [doi].

37. Bradbury K, Steele M, Corbett T, et al. Developing a digital intervention for cancer survivors: an evidence-, theory- and person-based approach. *NPJ digital medicine* 2019; 2. DOI: doi:10.1038/s41746-019-0163-4 [doi].

38. Brandon C, Doherty AJ, Kelly D, et al. HIPPP: health information portal for patients and public. *Applied Sciences* 2023; 13: 9453.

39. Bray L, Sharpe A, Gichuru P, et al. The Acceptability and Impact of the Xploro Digital Therapeutic Platform to Inform and Prepare Children for Planned Procedures in a Hospital: Before and After Evaluation Study. *Journal of medical Internet research* 2020; 22. DOI: doi:10.2196/17367 [doi].

40. Brown MC, Araújo-Soares V, Skinner R, et al. Using qualitative and co-design methods to inform the development of an intervention to support and improve physical activity in childhood cancer survivors: a study protocol for BEing Active after ChildhOod caNcer (BEACON). *BMJ open* 2020; 10. DOI: doi:10.1136/bmjopen-2020-041073 [doi].

41. Bruton A, Lee A, Yardley L, et al. Physiotherapy breathing retraining for asthma: a randomised controlled trial. *The Lancet Respiratory medicine* 2018; 6. DOI: doi:10.1016/S2213-2600(17)30474-5 [doi].

42. Castle EM, Dijk G, Asgari E, et al. The Feasibility and User-Experience of a Digital Health Intervention Designed to Prevent Weight Gain in New Kidney Transplant Recipients-The ExeRTiOn2 Trial. *Frontiers in nutrition* 2022; 9. DOI: doi:10.3389/fnut.2022.887580 [doi].

43. Chrétien-Vincent M, Grandisson M, Desmarais C, et al. Supporting the participation of autistic children: development, acceptability and feasibility of an intervention to build capacity of early childhood educators. *Journal of Early Childhood Teacher Education* 2023; 44: 1002-1023.

44. Clarkson P, Vassilev I, Rogers A, et al. Integrating a Web-Based Self-Management Tool (Managing Joint Pain on the Web and Through Resources) for People With Osteoarthritis-Related Joint Pain With a Web-Based Social Network Support Tool (Generating Engagement in Network Involvement): Design, Development, and Early Evaluation. *JMIR Form Res* 2020; 4: e18565. 20201126. DOI: 10.2196/18565.

45. Cong W, Chai J, Zhao L, et al. Cluster randomised controlled trial to assess a tailored intervention to reduce antibiotic prescribing in rural China: study protocol. *BMJ open* 2022; 12. DOI: doi:10.1136/bmjopen-2020-048267 [doi].

46. Cooray N, Sun SL, Ho C, et al. Toward a Behavior Theory-Informed and User-Centered Mobile App for Parents to Prevent Infant Falls: Development and Usability Study. *JMIR pediatrics and parenting* 2021; 4. DOI: doi:10.2196/29731 [doi].

47. Corbett T, Walsh JC, Groarke A, et al. Protocol for a pilot randomised controlled trial of an online intervention for post-treatment cancer survivors with persistent fatigue. *BMJ open* 2016; 6. DOI: doi:10.1136/bmjopen-2016-011485 [doi].

48. Corbett T, Walsh JC, Groarke A, et al. Cancer-related fatigue in post-treatment cancer survivors: theory-based development of a web-based intervention. *Jmir Cancer* 2017; 3: e6987.

49. Corbett TK, Cummings A, Lee K, et al. Planning and optimising CHAT&PLAN: A conversation-based intervention to promote person-centred care for older people living with multimorbidity. *PloS one* 2020; 15. DOI: doi:10.1371/journal.pone.0240516 [doi].

50. Cox JS, Searle A, Thornton G, et al. Integrating COM-B and the person-based approach to develop an ACT based therapy programme to raise self-determination in adolescents with obesity. *BMC Health Services Research* 2023; 23: 1158. DOI: 10.1186/s12913-023-09930-6.

51. Crane D, Garnett C, Brown J, et al. Factors influencing usability of a smartphone app to reduce excessive alcohol consumption: think aloud and interview studies. *Frontiers in public health* 2017; 5: 39.

52. Dale J, Loew J, Nanton V, et al. Coproduction of a Theory-Based Digital Resource for Unpaid Carers (The Care Companion): Mixed-Methods Study. *JMIR aging* 2018; 1. DOI: doi:10.2196/aging.9025 [doi].

53. Dale J, Nanton V, Day T, et al. Uptake and use of care companion, a web-based information resource for supporting informal carers of older people: mixed methods study. *JMIR aging* 2023; 6: e41185.

54. Dekker ARJ, Verheij TJM, Broekhuizen BDL, et al. Effectiveness of general practitioner online training and an information booklet for parents on antibiotic prescribing for children with respiratory tract infection in primary care: a cluster randomized controlled trial. *J Antimicrob Chemother* 2018; 73: 1416-1422. DOI: 10.1093/jac/dkx542.

55. Denecke K, Cihoric N and Reichenpfader D. Designing a digital medical interview assistant for radiology. *dHealth 2023* 2023: 60-66.

56. d'Offay C, Ng XY, Alexander L, et al. A digital health intervention for concussion: development and clinical feasibility study. *JMIR formative research* 2023; 7: e43557.

57. Dopke CA, McBride A, Babington P, et al. Development of Coaching Support for LiveWell: A Smartphone-Based Self-Management Intervention for Bipolar Disorder. *JMIR formative research* 2021; 5. DOI: doi:10.2196/25810 [doi].

58. Dougall G, Franssen M, Tucker KL, et al. Blood pressure monitoring in high-risk pregnancy to improve the detection and monitoring of hypertension (the BUMP 1 and 2 trials): protocol for two linked randomised controlled trials. *BMJ open* 2020; 10. DOI: doi:10.1136/bmjopen-2019-034593 [doi].

59. Drabble SJ, O'Cathain A, Scott AJ, et al. Mechanisms of Action of a Web-Based Intervention With Health Professional Support to Increase Adherence to Nebulizer Treatments in Adults With Cystic Fibrosis: Qualitative Interview Study. *Journal of medical Internet research* 2020; 22. DOI: doi:10.2196/16782 [doi].

60. Durrand J, Livingston R, Tew G, et al. Systematic development and feasibility testing of a multibehavioural digital prehabilitation intervention for patients approaching major surgery (iPREPWELL): A study protocol. *Plos one* 2022; 17: e0277143.

61. Easton S, Ainsworth B, Thomas M, et al. Planning a digital intervention for adolescents with asthma (BREATHE4T): A theory-, evidence- and Person-Based Approach to identify key behavioural issues. *Pediatric Pulmonology* 2022; 57. DOI: doi:https://dx.doi.org/10.1002/ppul.26099.

62. Eaves ER, Doerry E, Lanzetta SA, et al. Applying user-centered design in the development of a supportive mHealth app for women in substance use recovery. *American Journal of Health Promotion* 2023; 37: 56-64.

63. Essery R, Kirby S, Geraghty AW, et al. Older adults’ experiences of internet-based vestibular rehabilitation for dizziness: A longitudinal study. *Psychology & health* 2017; 32: 1327-1347.

64. Essery R, Denison-Day J, Grey E, et al. Development of the Digital Assessment of Precise Physical Activity (DAPPA) Tool for Older Adults. *International journal of environmental research and public health* 2020; 17. DOI: doi:10.3390/ijerph17217949 [doi].

65. Essery R, Pollet S, Smith KA, et al. Planning and optimising a digital intervention to protect older adults' cognitive health. *Pilot and feasibility studies* 2021; 7. DOI: doi:10.1186/s40814-021-00884-2 [doi].

66. Fareed N, Swoboda C, Singh P, et al. Developing and testing an integrated patient mHealth and provider dashboard application system for type 2 diabetes management among medicaid-enrolled pregnant individuals based on a user-centered approach: mixed-methods study. *Digital Health* 2023; 9: 20552076221144181.

67. Fisher H, Chantler T, Denford S, et al. Development of a multicomponent intervention to increase parental vaccine confidence and young people's access to the universal HPV vaccination programme in England: protocol for a co-design study. *BMJ open* 2022; 12. DOI: doi:10.1136/bmjopen-2022-062050 [doi].

68. Fisher H, Chantler T, Finn A, et al. Development of an educational package for the universal human papillomavirus (HPV) vaccination programme: a co-production study with young people and key informants. *Research involvement and engagement* 2022; 8. DOI: doi:10.1186/s40900-022-00349-7 [doi].

69. Flobak E, Nordby ES, Guribye F, et al. Designing Videos With and for Adults With ADHD for an Online Intervention: Participatory Design Study and Thematic Analysis of Evaluation. *JMIR mental health* 2021; 8. DOI: doi:10.2196/30292 [doi].

70. Furness PJ, Phelan I, Babiker NT, et al. Reducing pain during wound dressings in burn care using virtual reality: a study of perceived impact and usability with patients and nurses. *Journal of Burn Care & Research* 2019; 40: 878-885.

71. Gaffney H, Mansell W and Tai S. Agents of change: Understanding the therapeutic processes associated with the helpfulness of therapy for mental health problems with relational agent MYLO. *Digital health* 2020; 6. DOI: doi:10.1177/2055207620911580 [doi].

72. Gamble B, Depa K, Holmes EA, et al. Digitalizing a Brief Intervention to Reduce Intrusive Memories of Psychological Trauma: Qualitative Interview Study. *JMIR mental health* 2021; 8. DOI: doi:10.2196/23712 [doi].

73. Garnett C, Crane D, West R, et al. The development of Drink Less: an alcohol reduction smartphone app for excessive drinkers. *Translational behavioral medicine* 2019; 9. DOI: doi:10.1093/tbm/iby043 [doi].

74. Garnett C, Perski O, Michie S, et al. Refining the content and design of an alcohol reduction app, Drink Less, to improve its usability and effectiveness: a mixed methods approach. *F1000Research* 2021; 10.

75. Geraghty AWA, Stanford R, Little P, et al. Using an internet intervention to support self-management of low back pain in primary care: protocol for a randomised controlled feasibility trial (SupportBack). *BMJ open* 2015; 5. DOI: doi:10.1136/bmjopen-2015-009524 [doi].

76. Geraghty AW, Muñoz RF, Yardley L, et al. Developing an Unguided Internet-Delivered Intervention for Emotional Distress in Primary Care Patients: Applying Common Factor and Person-Based Approaches. *JMIR mental health* 2016; 3. DOI: doi:10.2196/mental.5845 [doi].

77. Geraghty AWA, Stanford R, Stuart B, et al. Using an internet intervention to support self-management of low back pain in primary care: findings from a randomised controlled feasibility trial (SupportBack). *BMJ open* 2018; 8. DOI: doi:10.1136/bmjopen-2017-016768 [doi].

78. Geraghty AW, Roberts LC, Stanford R, et al. Exploring patients’ experiences of internet-based self-management support for low back pain in primary care. *Pain Medicine* 2020; 21: 1806-1817.

79. Geraghty AWA, Roberts L, Hill J, et al. Supporting self-management of low back pain with an internet intervention in primary care: a protocol for a randomised controlled trial of clinical and cost-effectiveness (SupportBack 2). *BMJ open* 2020; 10. DOI: doi:10.1136/bmjopen-2020-040543 [doi].

80. Gilchrist G, Dheensa S, Johnson A, et al. Adapting the ADVANCE group program for digitally-supported delivery to reduce intimate partner violence by men in substance use treatment: a feasibility study. *Frontiers in Psychiatry* 2024; 14: 1253126. 20240124. DOI: 10.3389/fpsyt.2023.1253126.

81. Goddard SG, Stevens CJ and Swann C. Exploring runners’ perspectives of potential strategies for flow interventions. *Journal of Applied Sport Psychology* 2023; 35: 455-477.

82. Gonsalves PP, Hodgson ES, Kumar A, et al. Design and Development of the "POD Adventures" Smartphone Game: A Blended Problem-Solving Intervention for Adolescent Mental Health in India. *Frontiers in public health* 2019; 7. DOI: doi:10.3389/fpubh.2019.00238 [doi].

83. Gould R, Loebach Wetherell J, Serfaty M, et al. Acceptance and commitment therapy for older people with treatment-resistant generalised anxiety disorder: the FACTOID feasibility study. *Health Technology Assessment* 2021; 25.

84. Goulding EH, Dopke CA, Rossom RC, et al. A Smartphone-Based Self-management Intervention for Individuals With Bipolar Disorder (LiveWell): Empirical and Theoretical Framework, Intervention Design, and Study Protocol for a Randomized Controlled Trial. *JMIR research protocols* 2022; 11. DOI: doi:10.2196/30710 [doi].

85. Greenwell K, Sereda M, Coulson N, et al. Understanding User Reactions and Interactions With an Internet-Based Intervention for Tinnitus Self-Management: Mixed-Methods Process Evaluation Protocol. *JMIR research protocols* 2016; 5. DOI: doi:10.2196/resprot.5008 [doi].

86. Greenwell K, Sivyer K, Vedhara K, et al. Intervention planning for the REDUCE maintenance intervention: a digital intervention to reduce reulceration risk among patients with a history of diabetic foot ulcers. *BMJ open* 2018; 8. DOI: doi:10.1136/bmjopen-2017-019865 [doi].

87. Greenwell K, Sereda M, Coulson NS, et al. ‘That’s just how I am’: a qualitative interview study to identify factors influencing engagement with a digital intervention for tinnitus self‐management. *British Journal of Health Psychology* 2021; 26: 727-747.

88. Greenwell K, Ainsworth B, Bruton A, et al. Mixed methods process evaluation of my breathing matters, a digital intervention to support self-management of asthma. *NPJ primary care respiratory medicine* 2021; 31. DOI: doi:10.1038/s41533-021-00248-6 [doi].

89. Greenwell K, Sered M, Bradbury K, et al. Intervention planning for the tinnitus e-programme 2.0, an internet-based cognitive behavioral intervention for tinnitus. *American Journal of Audiology* 2021; 30. DOI: doi:10.1044/2021_AJA-20-00131.

90. Greenwell K, Ghio D, Sivyer K, et al. Eczema Care Online: development and qualitative optimisation of an online behavioural intervention to support self-management in young people with eczema. *BMJ open* 2022; 12. DOI: doi:10.1136/bmjopen-2021-056867 [doi].

91. Greville-Harris M, Bostock J, Din A, et al. Informing Patients About Placebo Effects: Using Evidence, Theory, and Qualitative Methods to Develop a New Website. *JMIR research protocols* 2016; 5. DOI: doi:10.2196/resprot.5627 [doi].

92. Gunn KM, Skaczkowski G, Dollman J, et al. Combining Farmers' Preferences With Evidence-Based Strategies to Prevent and Lower Farmers' Distress: Co-design and Acceptability Testing of ifarmwell. *JMIR human factors* 2022; 9. DOI: doi:10.2196/27631 [doi].

93. Hall K, Evans J, Roberts R, et al. Co-designing a nature-based intervention to promote postnatal mental health for mothers and their infants: a complex intervention development study in England. *BMJ open* 2023; 13: e075366.

94. Hashem F. MUSCLE STRENGTHENING INTERVENTION for BOYS with HAEMOPHILIA: DEVELOPING and EVALUATING A BEST-PRACTICE EXERCISE PROGRAMME with BOYS, FAMILIES and HEALTHCARE PROFESSIONALS. *Haemophilia* 2022; 28. DOI: doi:https://dx.doi.org/10.1111/hae.14477.

95. Hayes CV, Mahon B, Sides E, et al. Empowering Patients to Self-Manage Common Infections: Qualitative Study Informing the Development of an Evidence-Based Patient Information Leaflet. *Antibiotics (Basel, Switzerland)* 2021; 10. DOI: doi:10.3390/antibiotics10091113 [doi].

96. Heikkilä P, Mattila E and Ainasoja M. Designing a eustress toolbox: from entrepreneur experiences to an online service. *Human technology* 2018; 14: 233–257-233–257.

97. Hemmings NR, Kawadler JM, Whatmough R, et al. Development and Feasibility of a Digital Acceptance and Commitment Therapy-Based Intervention for Generalized Anxiety Disorder: Pilot Acceptability Study. *JMIR formative research* 2021; 5. DOI: doi:10.2196/21737 [doi].

98. Herbeć A, Perski O, Shahab L, et al. Smokers' Views on Personal Carbon Monoxide Monitors, Associated Apps, and Their Use: An Interview and Think-Aloud Study. *International journal of environmental research and public health* 2018; 15 20180207. DOI: doi:10.3390/ijerph15020288 [doi].

99. Heron N, O'Connor SR, Kee F, et al. Development of a Digital Lifestyle Modification Intervention for Use after Transient Ischaemic Attack or Minor Stroke: A Person-Based Approach. *International journal of environmental research and public health* 2021; 18. DOI: doi:10.3390/ijerph18094861 [doi].

100. Howarth A, Quesada J, Donnelly T, et al. The development of 'Make One Small Change': an e-health intervention for the workplace developed using the Person-Based Approach. *Digital health* 2019; 5. DOI: doi:10.1177/2055207619852856 [doi].

101. Hughes S, Kassianos AP, Everitt HA, et al. Planning and developing a web-based intervention for active surveillance in prostate cancer: an integrated self-care programme for managing psychological distress. *Pilot and feasibility studies* 2022; 8. DOI: doi:10.1186/s40814-022-01124-x [doi].

102. Ip A, Muller I, Geraghty AW, et al. Supporting Self-management Among Young People With Acne Vulgaris Through a Web-Based Behavioral Intervention: Development and Feasibility Randomized Controlled Trial. *JMIR dermatology* 2021; 4: e25918.

103. Jabban L, Metcalfe BW, Raines J, et al. Experience of adults with upper-limb difference and their views on sensory feedback for prostheses: a mixed methods study. *Journal of neuroengineering and rehabilitation* 2022; 19. DOI: doi:10.1186/s12984-022-01054-y [doi].

104. Jones RB, Merry S, Stallard P, et al. Further development and feasibility randomised controlled trial of a digital programme for adolescent depression, MoodHwb: study protocol. *BMJ open* 2023; 13: e070369.

105. Katzer CB, Mes MA, Chan AHY, et al. Acceptability of a theory-based adherence intervention for adults with asthma-a person-based approach. *Journal of Asthma* 2020; 57. DOI: doi:https://dx.doi.org/10.1080/02770903.2019.1609983.

106. Keenan E, Morris R, Vasiliou VS, et al. A qualitative feasibility and acceptability study of an acceptance and commitment-based bibliotherapy intervention for people with cancer. *Journal of Health Psychology* 2024; 29: 410-424.

107. Keller J, Roitzheim C, Radtke T, et al. A mobile intervention for self-efficacious and goal-directed smartphone use in the general population: randomized controlled trial. *JMIR mHealth and uHealth* 2021; 9: e26397.

108. Kelly PJ, Beck AK, Deane FP, et al. Feasibility of a Mobile Health App for Routine Outcome Monitoring and Feedback in SMART Recovery Mutual Support Groups: Stage 1 Mixed Methods Pilot Study. *Journal of medical Internet research* 2021; 23. DOI: doi:10.2196/25217 [doi].

109. Kendrick D, das Nair R, Kellezi B, et al. Vocational rehabilitation to enhance return to work after trauma (ROWTATE): protocol for a non-randomised single-arm mixed-methods feasibility study. *Pilot and feasibility studies* 2021; 7. DOI: doi:10.1186/s40814-021-00769-4 [doi].

110. Kenter RMF, Lundervold AJ and Nordgreen T. A self-guided Internet-delivered intervention for adults with ADHD: a protocol for a randomized controlled trial. *Internet interventions* 2021; 26. DOI: doi:10.1016/j.invent.2021.100485 [doi].

111. Kenter RMF, Schønning A and Inal Y. Internet-Delivered Self-help for Adults With ADHD (MyADHD): Usability Study. *JMIR formative research* 2022; 6. DOI: doi:10.2196/37137 [doi].

112. Kesten J, Hussey D, Lord C, et al. Development, acceptability and feasibility of a personalised, behavioural intervention to prevent bacterial skin and soft tissue infections among people who inject drugs: a mixed-methods Person-Based Approach study. *Harm Reduction Journal* 2023; 20: 114.

113. Kirwan R, Newson L, McCullough D, et al. Acceptability of a high-protein Mediterranean-style diet and resistance exercise protocol for cardiac rehabilitation patients: Involving service users in intervention design using a mixed-methods participatory approach. *Frontiers in Nutrition* 2023; 10: 1043391.

114. Knox L, Gemine R, Rees S, et al. Assessing the uptake, engagement, and safety of a self-management app, COPD. Pal®, for Chronic Obstructive Pulmonary Disease: a pilot study. *Health and Technology* 2021; 11: 557-562.

115. Korpershoek YJG, Hermsen S, Schoonhoven L, et al. User-Centered Design of a Mobile Health Intervention to Enhance Exacerbation-Related Self-Management in Patients With Chronic Obstructive Pulmonary Disease (Copilot): Mixed Methods Study. *Journal of medical Internet research* 2020; 22. DOI: doi:10.2196/15449 [doi].

116. Krishnaveni GV, Kumaran K, Krishna M, et al. Life course programming of stress responses in adolescents and young adults in India: Protocol of the Stress Responses in Adolescence and Vulnerability to Adult Non-communicable disease (SRAVANA) Study. *Wellcome open research* 2018; 3. DOI: doi:10.12688/wellcomeopenres.14583.1 [doi].

117. Kristjansdottir OB, Børøsund E, Westeng M, et al. Mobile App to Help People With Chronic Illness Reflect on Their Strengths: Formative Evaluation and Usability Testing. *JMIR formative research* 2020; 4. DOI: doi:10.2196/16831 [doi].

118. Krusche A, Bradbury K, Corbett T, et al. Renewed: Protocol for a randomised controlled trial of a digital intervention to support quality of life in cancer survivors. *BMJ open* 2019; 9. DOI: doi:10.1136/bmjopen-2018-024862 [doi].

119. Lawrason SVC, Brown-Ganzert L, Campeau L, et al. mHealth Physical Activity Intervention for Individuals With Spinal Cord Injury: Planning and Development Processes. *JMIR formative research* 2022; 6. DOI: doi:10.2196/34303 [doi].

120. Lawrence V, Kimona K, Howard RJ, et al. Optimising the acceptability and feasibility of acceptance and commitment therapy for treatment-resistant generalised anxiety disorder in older adults. *Age & Ageing* 2019; 48. DOI: doi:10.1093/ageing/afz082.

121. Learmonth YC, Adamson BC, Kinnett-Hopkins D, et al. Results of a feasibility randomised controlled study of the guidelines for exercise in multiple sclerosis project. *Contemporary clinical trials* 2017; 54: 84-97.

122. Lee C, Waite F, Piernas C, et al. Development and initial evaluation of a behavioural intervention to support weight management for people with serious mental illness: an uncontrolled feasibility and acceptability study. *BMC Psychiatry* 2023; 23: 130. DOI: 10.1186/s12888-023-04517-1.

123. Leupold F, Karimzadeh A, Breitkreuz T, et al. Digital redesign of hypertension management with practice and patient apps for blood pressure control (PIA study): A cluster-randomised controlled trial in general practices. *EClinicalMedicine* 2023; 55. DOI: doi:10.1016/j.eclinm.2022.101712 [doi].

124. Li X, Zhang Y, Ye Z, et al. Development of a Mobile Application of Internet-Based Support Program on Parenting Outcomes for Primiparous Women. *International journal of environmental research and public health* 2021; 18. DOI: doi:10.3390/ijerph18147354 [doi].

125. Little P, Stuart B, Hobbs FR, et al. Randomised controlled trial and economic analysis of an internet-based weight management programme: POWeR+ (Positive Online Weight Reduction). *Health technology assessment* 2017; 21.

126. Llahana S, Mulligan K, Hirani SP, et al. Using the behaviour change wheel and person-based approach to develop a digital self-management intervention for patients with adrenal insufficiency: the Support AI study protocol. *Frontiers in Endocrinology* 2023; 14: 1207715.

127. Llewelyn MJ, Budgell EP, Laskawiec-Szkonter M, et al. Antibiotic review kit for hospitals (ARK-Hospital): a stepped-wedge cluster-randomised controlled trial. *The Lancet Infectious Diseases* 2023; 23: 207-221.

128. Lown M, Smith KA, Muller I, et al. Internet tool to support self-assessment and self-swabbing of sore throat: development and feasibility study. *Journal of Medical Internet Research* 2023; 25: e39791.

129. Maenhout L, Peuters C, Cardon G, et al. Participatory development and pilot testing of an adolescent health promotion chatbot. *Frontiers in Public Health* 2021; 9: 724779.

130. Magin P, Tapley A, Morgan S, et al. Reducing early career general practitioners’ antibiotic prescribing for respiratory tract infections: a pragmatic prospective non-randomised controlled trial. *Family practice* 2018; 35: 53-60.

131. Mantzourani E, Brooks O, James D, et al. Development, implementation and evaluation of the digital transformation of renal services in Wales: the journey from local to national. *International journal of clinical pharmacy* 2022. DOI: doi:10.1007/s11096-022-01466-9 [doi].

132. Mathenjwa T, Kim H-Y, Zuma T, et al. Home-based intervention to test and start (HITS) protocol: a cluster-randomized controlled trial to reduce HIV-related mortality in men and HIV incidence in women through increased coverage of HIV treatment. *BMC public health* 2019; 19. DOI: doi:10.1186/s12889-019-7277-0 [doi].

133. Matthews L, Pugmire J, Moore L, et al. Study protocol for the 'HelpMeDoIt!' randomised controlled feasibility trial: an app, web and social support-based weight loss intervention for adults with obesity. *BMJ open* 2017; 7. DOI: doi:10.1136/bmjopen-2017-017159 [doi].

134. May T, Towler L, Smith LE, et al. Mpox knowledge, behaviours and barriers to public health measures among gay, bisexual and other men who have sex with men in the UK: a qualitative study to inform public health guidance and messaging. *BMC Public Health* 2023; 23: 2265.

135. McCaughan E, Flannagan C, Parahoo K, et al. The Tablet-Based, Engagement, Assessment, Support, and Sign-Posting (EASSi) Tool for Facilitating and Structuring Sexual Well-Being Conversations in Routine Prostate Cancer Care: Mixed-Methods Study. *JMIR Cancer* 2020; 6: e20137. 20201204. DOI: 10.2196/20137.

136. McDaid L, Emery J, Thomson R, et al. Development of “Baby, Me, & NRT”: A Behavioral Intervention to Improve the Effectiveness of Nicotine Replacement Therapy in Pregnancy. *Nicotine and Tobacco Research* 2023; 25: 1770-1780.

137. McDonald A. Art therapy for children following adverse childhood experiences: An intervention development study. *Arts in Psychotherapy* 2022; 77. DOI: doi:https://dx.doi.org/10.1016/j.aip.2022.101880.

138. McDonald B, Michelson D and Lester KJ. Intervention for school anxiety and absenteeism in children (ISAAC): Co-designing a brief parent-focused intervention for emotionally-based school avoidance. *Clinical child psychology and psychiatry* 2024; 29: 850-866.

139. McGrath N, Ngcobo N, Feng Z, et al. Protocol: evaluation of an optimised couples-focused intervention to increase testing for HIV in rural KwaZulu-Natal, South Africa, the Igugu Lethu ('Our treasure') cohort study. *BMC public health* 2022; 22. DOI: doi:10.1186/s12889-022-13894-3 [doi].

140. McKechnie AC, Elgersma KM, Iwaszko Wagner T, et al. An mHealth, patient engagement approach to understand and address parents' mental health and caregiving needs after prenatal diagnosis of critical congenital heart disease. *PEC Innov* 2023; 3: 100213. 20230909. DOI: 10.1016/j.pecinn.2023.100213.

141. McManus RJ, Little P, Stuart B, et al. Home and Online Management and Evaluation of Blood Pressure (HOME BP) using a digital intervention in poorly controlled hypertension: randomised controlled trial. *BMJ (Clinical research ed)* 2021; 372. DOI: doi:10.1136/bmj.m4858 [doi].

142. Mealer M, Conrad D, Evans J, et al. Feasibility and acceptability of a resilience training program for intensive care unit nurses. *American Journal of Critical Care* 2014; 23: e97-e105.

143. Mehra R, Pulman A, Dogan H, et al. A Tailored mHealth App for Improving Health and Well-Being Behavioral Transformation in UK Police Workers: Usability Testing via a Mixed Methods Study. *JMIR Hum Factors* 2023; 10: e42912. Original Paper 4.8.2023. DOI: 10.2196/42912.

144. Miller S, Ainsworth B, Weal M, et al. A Web-Based Intervention (Germ Defence) to Increase Handwashing During a Pandemic: Process Evaluations of a Randomized Controlled Trial and Public Dissemination. *Journal of medical Internet research* 2021; 23. DOI: doi:10.2196/26104 [doi].

145. Morris E, Aveyard P, Dyson P, et al. Dietary Approaches to the Management Of type 2 Diabetes (DIAMOND): protocol for a randomised feasibility trial. *BMJ open* 2019; 9. DOI: doi:10.1136/bmjopen-2018-026460 [doi].

146. Morris JH, Irvine LA, Dombrowski SU, et al. We Walk: a person-centred, dyadic behaviour change intervention to promote physical activity through outdoor walking after stroke-an intervention development study. *BMJ open* 2022; 12. DOI: doi:10.1136/bmjopen-2021-058563 [doi].

147. Morris RL, Giles S and Campbell S. Involving patients and carers in patient safety in primary care: A qualitative study of a co-designed patient safety guide. *Health Expect* 2023; 26: 630-639. 20230116. DOI: 10.1111/hex.13673.

148. Morrison D, Wyke S, Saunderson K, et al. Findings from a pilot Randomised trial of an Asthma Internet Self-management Intervention (RAISIN). *BMJ open* 2016; 6. DOI: doi:10.1136/bmjopen-2015-009254 [doi].

149. Morrison LG, Hargood C, Pejovic V, et al. The Effect of Timing and Frequency of Push Notifications on Usage of a Smartphone-Based Stress Management Intervention: An Exploratory Trial. *PloS one* 2017; 12. DOI: doi:10.1371/journal.pone.0169162 [doi].

150. Morton K, Dennison L, Bradbury K, et al. Qualitative process study to explore the perceived burdens and benefits of a digital intervention for self-managing high blood pressure in Primary Care in the UK. *BMJ open* 2018; 8. DOI: doi:10.1136/bmjopen-2017-020843 [doi].

151. Morton K, Dennison L, Band R, et al. Implementing a digital intervention for managing uncontrolled hypertension in Primary Care: a mixed methods process evaluation. *Implementation science : IS* 2021; 16. DOI: doi:10.1186/s13012-021-01123-1 [doi].

152. Morton K, Mhlakwaphalwa T, Msimango L, et al. Optimising a couples-focused intervention to increase couples' HIV testing and counselling using the person-based approach: a qualitative study in Kwa-Zulu Natal, South Africa. *BMJ open* 2021; 11. DOI: doi:10.1136/bmjopen-2020-047408 [doi].

153. Morton K, Towler L, Groot J, et al. Infection control in the home: a qualitative study exploring perceptions and experiences of adhering to protective behaviours in the home during the COVID-19 pandemic. *BMJ open* 2021; 11. DOI: doi:10.1136/bmjopen-2021-056161 [doi].

154. Morton K, Ainsworth B, Miller S, et al. Adapting Behavioral Interventions for a Changing Public Health Context: A Worked Example of Implementing a Digital Intervention During a Global Pandemic Using Rapid Optimisation Methods. *Front Public Health* 2021; 9: 668197. 20210426. DOI: 10.3389/fpubh.2021.668197.

155. Morton K, Kohut K, Turner L, et al. Person-based co-design of a decision aid template for people with a genetic predisposition to cancer. *Frontiers in Digital Health* 2022; 4: 1039701.

156. Mowbray F, Sivyer K, Santillo M, et al. Patient engagement with antibiotic messaging in secondary care: a qualitative feasibility study of the 'review and revise' experience. *Pilot and feasibility studies* 2020; 6. DOI: doi:10.1186/s40814-020-00590-5 [doi].

157. Mubangizi V, McGrath N, Kabakyenga JK, et al. Antenatal couples' counselling in Uganda (ACCU): study protocol for a randomised controlled feasibility trial. *Pilot and feasibility studies* 2022; 8. DOI: doi:10.1186/s40814-022-01049-5 [doi].

158. Mubangizi V, Plastow J, Nakaggwa F, et al. Assessing changes in knowledge, attitudes, and intentions to use family planning after watching documentary and drama health education films: a qualitative study. *Reproductive health* 2022; 19. DOI: doi:10.1186/s12978-022-01370-5 [doi].

159. Mueller J, Davies A, Jay C, et al. Developing and testing a web-based intervention to encourage early help-seeking in people with symptoms associated with lung cancer. *British journal of health psychology* 2019; 24. DOI: doi:10.1111/bjhp.12325 [doi].

160. Mueller J, Richards R, Jones RA, et al. Supporting Weight Management during COVID-19: A Randomized Controlled Trial of a Web-Based, ACT-Based, Guided Self-Help Intervention. *Obesity facts* 2022; 15. DOI: doi:10.1159/000524031 [doi].

161. Muir S, Newell C, Griffiths J, et al. MotivATE: A Pretreatment Web-Based Program to Improve Attendance at UK Outpatient Services Among Adults With Eating Disorders. *JMIR research protocols* 2017; 6. DOI: doi:10.2196/resprot.7440 [doi].

162. Muke SS, Tugnawat D, Joshi U, et al. Digital Training for Non-Specialist Health Workers to Deliver a Brief Psychological Treatment for Depression in Primary Care in India: Findings from a Randomized Pilot Study. *International journal of environmental research and public health* 2020; 17. DOI: doi:10.3390/ijerph17176368 [doi].

163. Muller I, Kirby S and Yardley L. Understanding patient experiences of self-managing chronic dizziness: a qualitative study of booklet-based vestibular rehabilitation, with or without remote support. *BMJ open* 2015; 5. DOI: doi:10.1136/bmjopen-2015-007680 [doi].

164. Muller I, Rowsell A, Stuart B, et al. Effects on Engagement and Health Literacy Outcomes of Web-Based Materials Promoting Physical Activity in People With Diabetes: An International Randomized Trial. *Journal of medical Internet research* 2017; 19. DOI: doi:10.2196/jmir.6601 [doi].

165. Muller I, Stuart B, Sach T, et al. Supporting self-care for eczema: protocol for two randomised controlled trials of ECO (Eczema Care Online) interventions for young people and parents/carers. *BMJ open* 2021; 11. DOI: doi:10.1136/bmjopen-2020-045583 [doi].

166. Murfield J, Moyle W and O'Donovan A. Planning and designing a self-compassion intervention for family carers of people living with dementia: a person-based and co-design approach. *BMC geriatrics* 2022; 22. DOI: doi:10.1186/s12877-022-02754-9 [doi].

167. Myklebost SB, Amundsen OM, Geraghty AWA, et al. Developing an internet-delivered intervention targeting residual cognitive symptoms after major depressive disorder: a person-based approach. *Journal of Mental Health* 2022. DOI: doi:https://dx.doi.org/10.1080/09638237.2021.2022618.

168. Naslund JA, Tyagi V, Khan A, et al. Schizophrenia Assessment, Referral and Awareness Training for Health Auxiliaries (SARATHA): protocol for a mixed-methods pilot study in rural India. *International Journal of Environmental Research and Public Health* 2022; 19: 14936.

169. Nevin SM, Wakefield CE, Dadich A, et al. Hearing parents' voices: a priority-setting workshop to inform a suite of psychological resources for parents of children with rare genetic epilepsies. *PEC innovation* 2022; 1: 100014.

170. Nevin SM, Wakefield CE, Le Marne F, et al. Piloting positive psychology resources for caregivers of a child with a genetic developmental and epileptic encephalopathy. *European Journal of Paediatric Neurology* 2022; 37: 129-138.

171. Nordby ES, Kenter RMF, Lundervold AJ, et al. A self-guided Internet-delivered intervention for adults with ADHD: A feasibility study. *Internet interventions* 2021; 25. DOI: doi:10.1016/j.invent.2021.100416 [doi].

172. Nordgreen T, Nordby ES, Myklebost SB, et al. In Case of an Emergency: The Development and Effects of a Digital Intervention for Coping With Distress in Norway During the COVID-19 Pandemic. *Frontiers in psychology* 2021; 12. DOI: doi:10.3389/fpsyg.2021.705383 [doi].

173. Notley C, Brown TJ, Bauld L, et al. Development of a Complex Intervention for the Maintenance of Postpartum Smoking Abstinence: Process for Defining Evidence-Based Intervention. *International journal of environmental research and public health* 2019; 16. DOI: doi:10.3390/ijerph16111968 [doi].

174. O'Connor SR, Flannagan C, Parahoo K, et al. Efficacy, Use, and Acceptability of a Web-Based Self-management Intervention Designed to Maximize Sexual Well-being in Men Living With Prostate Cancer: Single-Arm Experimental Study. *Journal of medical Internet research* 2021; 23. DOI: doi:10.2196/21502 [doi].

175. Ong SW, Wong JV, Auguste BL, et al. Design and development of a digital counseling program for chronic kidney disease. *Canadian Journal of Kidney Health and Disease* 2022; 9: 20543581221103683.

176. Payne L, Harris P, Ghio D, et al. Beliefs about inevitable decline among home-living older adults at risk of malnutrition: a qualitative study. *Journal of human nutrition and dietetics : the official journal of the British Dietetic Association* 2020; 33. DOI: doi:https://dx.doi.org/10.1111/jhn.12807.

177. Payne L, Ghio D, Grey E, et al. Optimising an intervention to support home-living older adults at risk of malnutrition: a qualitative study. *BMC family practice* 2021; 22. DOI: doi:10.1186/s12875-021-01572-z [doi].

178. Pegington M, Davies A, Mueller J, et al. Evaluating the acceptance and usability of an app promoting weight gain prevention and healthy behaviors among young women with a family history of breast cancer: protocol for an observational study. *JMIR research protocols* 2022; 11: e41246.

179. Pinto da Costa M. An Intervention to Connect Patients With Psychosis and Volunteers via Smartphone (the Phone Pal): Development Study. *JMIR formative research* 2022; 6. DOI: doi:10.2196/35086 [doi].

180. Pinto C, Geraghty AW, Pagnini F, et al. How do people with MND and caregivers experience a digital mental health intervention? A qualitative study. *Frontiers in Psychiatry* 2023; 14: 1083196.

181. Pollet S, Denison-Day J, Bradbury K, et al. A Qualitative Exploration of Perceptions of a Digital Intervention to Promote Physical Activity in Older Adults. *Journal of aging and physical activity* 2020; 29. DOI: doi:https://dx.doi.org/10.1123/japa.2019-0484.

182. Poppe L, De Bourdeaudhuij I, Verloigne M, et al. A Self-Regulation-Based eHealth and mHealth Intervention for an Active Lifestyle in Adults With Type 2 Diabetes: Protocol for a Randomized Controlled Trial. *JMIR research protocols* 2019; 8. DOI: doi:10.2196/12413 [doi].

183. Porritt J, Rodd H, Morgan A, et al. Development and Testing of a Cognitive Behavioral Therapy Resource for Children's Dental Anxiety. *JDR clinical and translational research* 2017; 2. DOI: doi:10.1177/2380084416673798 [doi].

184. Rai T, Morton K, Roman C, et al. Optimizing a digital intervention for managing blood pressure in stroke patients using a diverse sample: Integrating the person-based approach and patient and public involvement. *Health expectations : an international journal of public participation in health care and health policy* 2021; 24. DOI: doi:10.1111/hex.13173 [doi].

185. Reale S, Turner RR, Sutton E, et al. Towards implementing exercise into the prostate cancer care pathway: development of a theory and evidence-based intervention to train community-based exercise professionals to support change in patient exercise behaviour (The STAMINA trial). *BMC Health Serv Res* 2021; 21: 264. 20210322. DOI: 10.1186/s12913-021-06275-w.

186. Remskar M, Atkinson MJ, Marks E, et al. Understanding university student priorities for mental health and well‐being support: A mixed‐methods exploration using the person‐based approach. *Stress & Health: Journal of the International Society for the Investigation of Stress* 2022; 38. DOI: doi:10.1002/smi.3133.

187. Rhodes A, Pimprikar A, Baum A, et al. Using the Person-Based Approach to Develop a Digital Intervention Targeting Diet and Physical Activity in Pregnancy: Development Study. *JMIR Form Res* 2023; 7: e44082. 20230526. DOI: 10.2196/44082.

188. Richards R, Kinnersley P, Brain K, et al. Cancer Clinicians' Views Regarding an App That Helps Patients With Cancer Meet Their Information Needs: Qualitative Interview Study. *JMIR cancer* 2021; 7. DOI: doi:10.2196/23671 [doi].

189. Richards R, Jones RA, Whittle F, et al. Development of a Web-Based, Guided Self-help, Acceptance and Commitment Therapy-Based Intervention for Weight Loss Maintenance: Evidence-, Theory-, and Person-Based Approach. *JMIR formative research* 2022; 6: e31801. 20220107. DOI: doi:10.2196/31801 [doi].

190. Rosenthal JL, Sauers-Ford HS, Hamline MY, et al. Developing an interfacility transfer handoff intervention: applying the person-based approach method. *Hospital Pediatrics* 2020; 10: 577-584.

191. Rowsell A, Muller I, Murray E, et al. Views of People With High and Low Levels of Health Literacy About a Digital Intervention to Promote Physical Activity for Diabetes: A Qualitative Study in Five Countries. *Journal of medical Internet research* 2015; 17. DOI: doi:10.2196/jmir.4999 [doi].

192. Santer M, Muller I, Becque T, et al. Eczema Care Online behavioural interventions to support self-care for children and young people: two independent, pragmatic, randomised controlled trials. *bmj* 2022; 379.

193. Santillo M, Sivyer K, Krusche A, et al. Intervention planning for Antibiotic Review Kit (ARK): a digital and behavioural intervention to safely review and reduce antibiotic prescriptions in acute and general medicine. *The Journal of antimicrobial chemotherapy* 2019; 74. DOI: doi:10.1093/jac/dkz333 [doi].

194. Saxton JM, Pickering K, Wane S, et al. Co-designed weight management intervention for women recovering from oestrogen-receptor positive breast cancer. *BMC cancer* 2022; 22. DOI: doi:10.1186/s12885-022-10287-y [doi].

195. Schaab BL and Remor E. Development, feasibility testing and perceived benefits of a new app to help with adherence to antiretroviral therapy in people living with HIV in Brazil. *Pilot Feasibility Stud* 2023; 9: 130. 20230726. DOI: 10.1186/s40814-023-01370-7.

196. Scholefield BR, Menzies JC, McAnuff J, et al. Implementing early rehabilitation and mobilisation for children in UK paediatric intensive care units: the PERMIT feasibility study. *Health Technology Assessment (Winchester, England)* 2023; 27: 1. DOI: 10.3310/HYRW5688.

197. Scott J, Oxlad M, Dodd J, et al. Promoting Health Behavior Change in the Preconception Period: Combined Approach to Intervention Planning. *JMIR formative research* 2022; 6. DOI: doi:10.2196/35108 [doi].

198. Seaton N, Moss-Morris R, Hulme K, et al. A cognitive–behavioural therapy programme for managing depression and anxiety in long-term physical health conditions: mixed-methods real-world evaluation of the COMPASS programme. *BJPsych Open* 2023; 9: e153.

199. Sekse RJT, Nordgreen T, Flobak E, et al. Development of a Framework and the Content for a Psychoeducational Internet-Delivered Intervention for Women after Treatment for Gynecological Cancer. *Nursing reports (Pavia, Italy)* 2021; 11. DOI: doi:10.3390/nursrep11030061 [doi].

200. Semple CJ and McCaughan E. Developing and testing a theory-driven e-learning intervention to equip healthcare professionals to communicate with parents impacted by parental cancer. *European Journal of Oncology Nursing* 2019; 41. DOI: doi:10.1016/j.ejon.2019.05.006.

201. Semple C, Kelly R, Thompson R, et al. Informing the development of an online resource for patients with oral cancer: triangulation of qualitative data from patients and healthcare professionals. *Frontiers of Oral and Maxillofacial Medicine* 2022: 1-15.

202. Sezier A, Mudge S, Kayes N, et al. Development of a toolkit to enhance care processes for people with a long-term neurological condition: a qualitative descriptive study. *BMJ open* 2018; 8. DOI: doi:10.1136/bmjopen-2018-022038 [doi].

203. Sharman LS, Avent ML, Lyall V, et al. Improving paediatric antimicrobial stewardship in remote and regional Queensland hospitals: development and qualitative evaluation of a tailored intervention for intravenous-to-oral antibiotic switching. *BMJ open* 2022; 12: e064888.

204. Shaw S, Simao SC, Jenner S, et al. Parental perspectives on negotiations over diet and physical activity: how do we involve parents in adolescent health interventions? *Public Health Nutrition* 2021; 24: 2727-2736.

205. Shoneye CL, Mullan B, Begley A, et al. Design and Development of a Digital Weight Management Intervention (ToDAy): Qualitative Study. *JMIR mHealth and uHealth* 2020; 8. DOI: doi:10.2196/17919 [doi].

206. Singh L, Kanstrup M, Depa K, et al. Digitalizing a Brief Intervention to Reduce Intrusive Memories of Psychological Trauma for Health Care Staff Working During COVID-19: Exploratory Pilot Study With Nurses. *JMIR formative research* 2021; 5. DOI: doi:10.2196/27473 [doi].

207. Sivyer K, Teasdale E, Greenwell K, et al. Supporting families managing childhood eczema: developing and optimising eczema care online using qualitative research. *The British journal of general practice : the journal of the Royal College of General Practitioners* 2022; 72. DOI: doi:10.3399/BJGP.2021.0503 [doi].

208. Smith E, Bradbury K, Scott L, et al. Providing online weight management in Primary Care: a mixed methods process evaluation of healthcare practitioners' experiences of using and supporting patients using POWeR. *Implementation science : IS* 2017; 12. DOI: doi:10.1186/s13012-017-0596-6 [doi].

209. Smith A, Bamgboje-Ayodele A, Butow P, et al. Development and usability evaluation of an online self-management intervention for fear of cancer recurrence (iConquerFear). *Psycho-Oncology* 2020; 29. DOI: doi:10.1002/pon.5218.

210. Smith KA, Vennik J, Morrison L, et al. Harnessing Placebo Effects in Primary Care: Using the Person-Based Approach to Develop an Online Intervention to Enhance Practitioners' Communication of Clinical Empathy and Realistic Optimism During Consultations. *Frontiers in pain research (Lausanne, Switzerland)* 2021; 2. DOI: doi:10.3389/fpain.2021.721222 [doi].

211. Smith KA, Van Pinxteren M, Mbokazi N, et al. Intervention development of ‘Diabetes Together’using the person-based approach: a couples-focused intervention to support self-management of type 2 diabetes in South Africa. *BMJ open* 2023; 13: e069982.

212. Stavric V, Saywell N and Kayes NM. Development of a self-guided web-based exercise intervention (SPIN) to treat shoulder pain in people living with spinal cord injury: protocol of a mixed methods study. *BMJ open* 2019; 9. DOI: doi:10.1136/bmjopen-2019-031012 [doi].

213. Stavric V, Saywell NL and Kayes NM. Perceptions of a self-guided web-based exercise programme for shoulder pain after spinal cord injury: A qualitative study. *Spinal Cord* 2023; 61: 238-243.

214. Steed L, Heslop-Marshall K, Sohanpal R, et al. Developing a complex intervention whilst considering implementation: the TANDEM (Tailored intervention for ANxiety and DEpression Management) intervention for patients with chronic obstructive pulmonary disease (COPD). *Trials* 2021; 22. DOI: doi:10.1186/s13063-021-05203-x [doi].

215. Steed L, Sheringham J, McClatchey K, et al. IMP2ART: development of a multi-level programme theory integrating the COM-B model and the iPARIHS framework, to enhance implementation of supported self-management of asthma in primary care. *Implementation Science Communications* 2023; 4: 136.

216. Strömmer S, Barrett M, Woods-Townsend K, et al. Engaging adolescents in changing behaviour (EACH-B): a study protocol for a cluster randomised controlled trial to improve dietary quality and physical activity. *Trials* 2020; 21. DOI: doi:10.1186/s13063-020-04761-w [doi].

217. Sugg HVR, Richards DA and Frost J. Optimising the acceptability and feasibility of novel complex interventions: an iterative, person-based approach to developing the UK Morita therapy outpatient protocol. *Pilot and feasibility studies* 2017; 3. DOI: doi:10.1186/s40814-017-0181-4 [doi].

218. Swanston E, Pulman A, Dogan H, et al. Scoping the Need for a Tailored mHealth App to Improve Health and Well-being Behavioral Transformation in the Police: Exploring the Views of UK Police Workers via Web-Based Surveys and Client Meetings. *JMIR formative research* 2021; 5. DOI: doi:10.2196/28075 [doi].

219. Sweeney L, Windgassen S, Artom M, et al. A Novel Digital Self-management Intervention for Symptoms of Fatigue, Pain, and Urgency in Inflammatory Bowel Disease: Describing the Process of Development. *JMIR formative research* 2022; 6. DOI: doi:10.2196/33001 [doi].

220. Tatar O, Abdel-Baki A, Dyachenko A, et al. Evaluating preferences for online psychological interventions to decrease cannabis use in young adults with psychosis: An observational study. *Psychiatry research* 2023; 326: 115276. 20230530. DOI: 10.1016/j.psychres.2023.115276.

221. Thomas M, Bruton A, Little P, et al. A randomised controlled study of the effectiveness of breathing retraining exercises taught by a physiotherapist either by instructional DVD or in face-to-face sessions in the management of asthma in adults. *Health Technology Assessment* 2017; 21: 1-161.

222. Thompson K, Milligan J, Briggs M, et al. A qualitative study to explore the acceptability and feasibility of implementing person-focused evidence-based pain education concepts in pre-registration physiotherapy training. *Frontiers in Pain Research* 2023; 4: 1162387.

223. Tindall T, Topcu G, Thomas S, et al. Developing a patient care pathway for emotional support around the point of multiple sclerosis diagnosis: A stakeholder engagement study. *Health Expect* 2023; 26: 858-868. 20230123. DOI: 10.1111/hex.13711.

224. Tinner LE, Kaner E, Garnett C, et al. Qualitative Evaluation of Web-Based Digital Intervention to Prevent and Reduce Excessive Alcohol Use and Harm Among Young People Aged 14-15 Years: A "Think-Aloud" Study. *JMIR Pediatr Parent* 2020; 3: e19749. 20201215. DOI: 10.2196/19749.

225. Topcu G, Buchanan H, Aubeeluck A, et al. Informal carers’ experiences of caring for someone with Multiple Sclerosis: A photovoice investigation. *British journal of health psychology* 2021; 26: 360-384.

226. Treneman-Evans G, Ali B, Denison-Day J, et al. The Rapid Adaptation and Optimisation of a Digital Behaviour-Change Intervention to Reduce the Spread of COVID-19 in Schools. *International journal of environmental research and public health* 2022; 19. DOI: doi:10.3390/ijerph19116731 [doi].

227. Tudor K, Brooks J, Howick J, et al. Tackling statin intolerance with n-of-1 trials (TaSINI) in primary care: protocol for a feasibility randomised trial to increase statin adherence. *BMJ open* 2020; 10. DOI: doi:10.1136/bmjopen-2019-033070 [doi].

228. Tudor K, Brooks J, Howick J, et al. Unblinded and Blinded N-of-1 Trials Versus Usual Care: A Randomized Controlled Trial to Increase Statin Uptake in Primary Care. *Circulation: Cardiovascular Quality and Outcomes* 2022; 15: e007793. DOI: doi:10.1161/CIRCOUTCOMES.120.007793.

229. Vajravelu ME, Hitt TA, Mak N, et al. Text Messages and Financial Incentives to Increase Physical Activity in Adolescents With Prediabetes and Type 2 Diabetes: Web-Based Group Interviews to Inform Intervention Design. *JMIR diabetes* 2022; 7. DOI: doi:10.2196/33082 [doi].

230. van Vugt VA, van der Wouden JC, Essery R, et al. Internet based vestibular rehabilitation with and without physiotherapy support for adults aged 50 and older with a chronic vestibular syndrome in general practice: three armed randomised controlled trial. *BMJ (Clinical research ed)* 2019; 367. DOI: doi:10.1136/bmj.l5922 [doi].

231. Vennik J, Geraghty AW, Martinson K, et al. Determining the clinical and cost-effectiveness of nasal sprays and a physical activity and stress management intervention to reduce respiratory tract infections in primary care: A protocol for the ‘Immune Defence’randomised controlled trial. *Plos one* 2023; 18: e0285693.

232. Warne E, Oxlad M and Best T. Consulting patients and providers of assisted reproductive technologies to inform the development of a group psychological intervention for women with infertility. *PEC innovation* 2023; 3: 100206. 20230830. DOI: 10.1016/j.pecinn.2023.100206.

233. Watland S, Solberg Nes L, Hanson E, et al. The Caregiver Pathway, a Model for the Systematic and Individualized Follow-up of Family Caregivers at Intensive Care Units: Development Study. *JMIR Form Res* 2023; 7: e46299. 20230425. DOI: 10.2196/46299.

234. Weerasekara M, Smedberg ÅB, Karunathilaka G, et al. User needs gathering for the design of information and communications technology-supported occupational stress management intervention: A quantitative study. *Digital health* 2022; 8. DOI: doi:10.1177/20552076221127778 [doi].

235. Whale K, Beasant L, Wright AJ, et al. A smartphone app for supporting the self-management of daytime urinary incontinence in adolescents: development and formative evaluation study of URApp. *JMIR pediatrics and parenting* 2021; 4: e26212.

236. Wildman MJ, O’Cathain A, Hind D, et al. An intervention to support adherence to inhaled medication in adults with cystic fibrosis: the ACtiF research programme including RCT. *Programme Grants for Applied Research* 2021; 9: 1-146.

237. Williamson S, Dennison L, Greenwell K, et al. Using nasal sprays to prevent respiratory tract infections: a qualitative study of online consumer reviews and primary care patient interviews. *BMJ open* 2022; 12. DOI: doi:10.1136/bmjopen-2021-059661 [doi].

238. Wuyts M, Hermans F, Breuls S, et al. Development and feasibility of an exercise training program in primary care for patients with COPD experiencing an acute exacerbation. *Physiotherapy* 2024; 123: 81-90.

239. Zamanillo-Campos R, Fiol-deRoque MA, Serrano-Ripoll MJ, et al. Development and evaluation of DiabeText, a personalized mHealth intervention to support medication adherence and lifestyle change behaviour in patients with type 2 diabetes in Spain: A mixed-methods phase II pragmatic randomized controlled clinical trial. *International Journal of Medical Informatics* 2023; 176: 105103.
