# Supplementary material for: The person-based approach to intervention development: A scoping review of methods and applications
Source: Digit Health. 2025 Jan 9;11:20552076241305934. doi: 10.1177/20552076241305934 (PMC11719439; doi:10.1177/20552076241305934)
Supplement: sj-docx-3-dhj-10.1177_20552076241305934 - Supplemental material for The person-based approach to intervention development: A scoping review of methods and applications [file sj-docx-3-dhj-10.1177_20552076241305934.docx]

Supplementary Material

#### Codebook

| Name | Description and example |
| --- | --- |
| **Intervention focused** | Using the PBA to create content which could establish positive user interactions with intervention content, in order to reach successful outcomes. |
| acceptable | “…to ensure that the intervention is effective and acceptable to those who will ultimately use it”^1^ |
| accessible | “…to co-design (with patients) an accessible and adoptable weight loss intervention”^2^ |
| effective | “This helped us to create an intervention relevant for people with SMI and thereby improve the likelihood of it being effective”^3^ |
| engaging | “This perspective is also recommended in the person-based approach to developing usable and engaging health related interventions"^4^ |
| feasible | “This approach allows designers to understand what to do to design an attractive intervention that (1) addresses end users’ needs and (2) is feasible to implement”^5^ |
| persuasive | “…aims to optimize the design of complex interventions and ensure they are not just acceptable and usable but also engaging and persuasive”^6^ |
| **Methods focused** | A proven, rigorous and systematic process for intervention design which guides researchers in the use of specific methods |
| evidence | “…incorporated the perspectives of carers by synthesizing evidence from the research and policy literature, with active input from carers and stakeholders throughout the developmental process”^7^ |
| feedback | “…by continuing to incorporate user feedback after live testing of the intervention”^8^ |
| guiding principles | “…provides a systematic method to apply this gained understanding, by developing guiding principles and key intervention features to optimise intervention benefits.”^8^ |
| iterative | “Importantly, each step was used iteratively to inform the next step with repeated cycling between steps where needed.”^9^ |
| mixed-methods | “…which accounts for the context, perspectives, and experiences of end users through mixed methods research”^10^ |
| optimize | “We optimised the intervention tools using qualitative research to identify barriers to acceptability, feasibility and engagement that need to be addressed"^11^ |
| participatory | “We drew on the PBA for its suitability for designing interventions with digital components and focus on stakeholder engagement and co-design with target users”^12^ |
| process oriented | “…provides a systematic method to apply this gained understanding, by developing guiding principles and key intervention features to optimise intervention benefits”^13^ |
| qualitative research | “…to supplement existing evidence and theory with new primary qualitative research with important stakeholders and can help define relevant components of an intervention in more detail”^13^ |
| rigorous | “…the person-based approach provided a systematic and robust method to combine user-centred design methods with evidence-based behaviour change methods."^14^ |
| successful method | “…it has been used to develop several effective and cost-effective behavioural interventions to help manage various long-term health conditions”^15^ |
| theory | “…to ensure that the resulting intervention is systematically developed from the bottom- up and is theory- informed and evidence- based"^16^ |
| think-alouds | “…user testing is an important part of the intervention development process, and think-aloud studies are commonly used for this purpose”^17^ |
| **User focused** | A way to understand user needs and preferences at the interface of the intervention, as well as their wider psychosocial contexts. |
| user involvement | “…emphasizing the importance of end-user involvement and iterative testing to promote acceptability and effectiveness”^18^ |
| user context | “…The person-based approach allowed an in-depth understanding of the biopsychosocial context of caring..”^7^ |
| user experience | “…an important part of person-based intervention development as it helps in understanding participants’ experiences and acceptability of the therapy”^19^ |
| user understanding | “…We used a human-centered approach to optimize the understanding and accommodate the perspectives of potential users”^20^ |

1. Clarkson P, Vassilev I, Rogers A, et al. Integrating a Web-Based Self-Management Tool (Managing Joint Pain on the Web and Through Resources) for People With Osteoarthritis-Related Joint Pain With a Web-Based Social Network Support Tool (Generating Engagement in Network Involvement): Design, Development, and Early Evaluation. *JMIR formative research* 2020; 4. DOI: doi:10.2196/18565 [doi].

2. Saxton JM, Pickering K, Wane S, et al. Co-designed weight management intervention for women recovering from oestrogen-receptor positive breast cancer. *BMC cancer* 2022; 22. DOI: doi:10.1186/s12885-022-10287-y [doi].

3. Lee C, Waite F, Piernas C, et al. Development and initial evaluation of a behavioural intervention to support weight management for people with serious mental illness: an uncontrolled feasibility and acceptability study. *BMC Psychiatry* 2023; 23: 130. DOI: 10.1186/s12888-023-04517-1.

4. Sekse RJT, Nordgreen T, Flobak E, et al. Development of a Framework and the Content for a Psychoeducational Internet-Delivered Intervention for Women after Treatment for Gynecological Cancer. *Nursing reports (Pavia, Italy)* 2021; 11. DOI: doi:10.3390/nursrep11030061 [doi].

5. Ardern CL, Hooper N, O'Halloran P, et al. A Psychological Support Intervention to Help Injured Athletes "Get Back in the Game": Design and Development Study. *JMIR formative research* 2022; 6. DOI: doi:10.2196/28851 [doi].

6. McDonald A. Art therapy for children following adverse childhood experiences: An intervention development study. *Arts in Psychotherapy* 2022; 77. DOI: doi:<https://dx.doi.org/10.1016/j.aip.2022.101880>.

7. Dale J, Loew J, Nanton V, et al. Coproduction of a Theory-Based Digital Resource for Unpaid Carers (The Care Companion): Mixed-Methods Study. *JMIR aging* 2018; 1. DOI: doi:10.2196/aging.9025 [doi].

8. Jabban L, Metcalfe BW, Raines J, et al. Experience of adults with upper-limb difference and their views on sensory feedback for prostheses: a mixed methods study. *Journal of neuroengineering and rehabilitation* 2022; 19. DOI: doi:10.1186/s12984-022-01054-y [doi].

9. Li X, Zhang Y, Ye Z, et al. Development of a Mobile Application of Internet-Based Support Program on Parenting Outcomes for Primiparous Women. *International journal of environmental research and public health* 2021; 18. DOI: doi:10.3390/ijerph18147354 [doi].

10. Lawrason SVC, Brown-Ganzert L, Campeau L, et al. mHealth Physical Activity Intervention for Individuals With Spinal Cord Injury: Planning and Development Processes. *JMIR formative research* 2022; 6. DOI: doi:10.2196/34303 [doi].

11. Cong W, Chai J, Zhao L, et al. Cluster randomised controlled trial to assess a tailored intervention to reduce antibiotic prescribing in rural China: study protocol. *BMJ open* 2022; 12. DOI: doi:10.1136/bmjopen-2020-048267 [doi].

12. Borek AJ, Campbell A, Dent E, et al. Development of an intervention to support the implementation of evidence-based strategies for optimising antibiotic prescribing in general practice. *Implementation science communications* 2021; 2. DOI: doi:10.1186/s43058-021-00209-7 [doi].

13. Katzer CB, Mes MA, Chan AHY, et al. Acceptability of a theory-based adherence intervention for adults with asthma-a person-based approach. *Journal of Asthma* 2020; 57. DOI: doi:<https://dx.doi.org/10.1080/02770903.2019.1609983>.

14. Bingham SL, Semple CJ, Flannagan C, et al. Adapting and usability testing of an eLearning resource to enhance healthcare professional provision of sexual support across cancer care. *Supportive care in cancer : official journal of the Multinational Association of Supportive Care in Cancer* 2022; 30. DOI: doi:10.1007/s00520-022-06798-w [doi].

15. Easton S, Ainsworth B, Thomas M, et al. Planning a digital intervention for adolescents with asthma (BREATHE4T): A theory-, evidence- and Person-Based Approach to identify key behavioural issues. *Pediatric Pulmonology* 2022; 57. DOI: doi:<https://dx.doi.org/10.1002/ppul.26099>.

16. Brown MC, Araújo-Soares V, Skinner R, et al. Using qualitative and co-design methods to inform the development of an intervention to support and improve physical activity in childhood cancer survivors: a study protocol for BEing Active after ChildhOod caNcer (BEACON). *BMJ open* 2020; 10. DOI: doi:10.1136/bmjopen-2020-041073 [doi].

17. Cooray N, Sun SL, Ho C, et al. Toward a Behavior Theory-Informed and User-Centered Mobile App for Parents to Prevent Infant Falls: Development and Usability Study. *JMIR pediatrics and parenting* 2021; 4. DOI: doi:10.2196/29731 [doi].

18. Semple CJ and McCaughan E. Developing and testing a theory-based e-learning intervention to enhance healthcare professional's self-efficacy when supporting parents newly diagnosed with cancer who have dependent children. *Annals of Oncology* 2018; 29. DOI: doi:<https://dx.doi.org/10.1093/annonc/mdy276.021>.

19. Muller I, Kirby S and Yardley L. Understanding patient experiences of self-managing chronic dizziness: a qualitative study of booklet-based vestibular rehabilitation, with or without remote support. *BMJ open* 2015; 5. DOI: doi:10.1136/bmjopen-2015-007680 [doi].

20. Swanston E, Pulman A, Dogan H, et al. Scoping the Need for a Tailored mHealth App to Improve Health and Well-being Behavioral Transformation in the Police: Exploring the Views of UK Police Workers via Web-Based Surveys and Client Meetings. *JMIR formative research* 2021; 5. DOI: doi:10.2196/28075 [doi].
